# Microencapsulation of Probiotics for Enhanced Stability and Health Benefits in Dairy Functional Foods: A Focus on Pasta Filata Cheese

**DOI:** 10.3390/pharmaceutics17020185

**Published:** 2025-02-02

**Authors:** Vita D’Amico, Mariasimona Cavaliere, Marianna Ivone, Chiara Lacassia, Giuseppe Celano, Mirco Vacca, Flavia Maria la Forgia, Sergio Fontana, Maria De Angelis, Nunzio Denora, Angela Assunta Lopedota

**Affiliations:** 1 Department of Pharmacy—Pharmaceutical Sciences, University of Bari Aldo Moro, 4, E. Orabona Street, 70125 Bari, Italy; vita.damico@uniba.it (V.D.); marianna.ivone@uniba.it (M.I.); chiara.lacassia@uniba.it (C.L.); nunzio.denora@uniba.it (N.D.); 2 Department of Soil, Plant and Food Sciences, University of Bari Aldo Moro, 165/A, G. Amendola Street, 70126 Bari, Italy; m.cavaliere14@phd.uniba.it (M.C.); giuseppe.celano@uniba.it (G.C.); mirco.vacca@uniba.it (M.V.); maria.deangelis@uniba.it (M.D.A.); 3 Centro Studi e Ricerche “Dr. S. Fontana 1900–1982”, Farmalabor s.r.l., 47, Piano S. Giovanni Street, 76012 Canosa di Puglia, Italy; f.laforgia@farmalabor.it (F.M.l.F.); s.fontana@farmalabor.it (S.F.)

**Keywords:** probiotics, prebiotics, microencapsulation techniques, dairy functional foods, pasta filata, enhanced cell viability, synbiotics

## Abstract

Probiotics provide significant health benefits, but their viability is often compromised during production, storage, and passage through the gastrointestinal tract. These challenges hinder their effective incorporation into functional applications, particularly in dairy functional foods, in which factors such as acidity, oxygen exposure, and storage conditions negatively impact cell survival. The focus was on functional dairy foods, particularly on pasta filata cheeses. Indeed, the use of probiotics in pasta filata cheeses presents significant challenges due to the specific manufacturing processes, which encompass the application of high temperatures and other harsh conditions. These factors can adversely affect the viability and availability of probiotic microorganisms. However, microencapsulation has emerged as a promising solution, offering a protective barrier that enhances probiotic stability, improves survival rates, and facilitates targeted release in the gastrointestinal environment. This review examines the pivotal role of microencapsulation in stabilising probiotics for functional applications, emphasising its relevance in high-value food systems. Functional applications, including foods designed to offer essential nutritional benefits and promote host health, play a crucial role in disease prevention and immune system support, reducing the risk of infections and other physiological impairments. Key microencapsulation technologies are analysed, focusing on their benefits, limitations, and challenges related to scalability and industrial implementation. Additionally, this review discusses strategies to optimise formulations, ensure the sensory quality of final products, and explore future opportunities for expanding innovative applications that align with growing consumer demand for health-promoting solutions.

## 1. Introduction

### 1.1. Definition of Probiotics

Over time, researchers have discovered that certain bacteria can positively influence intestinal microbiota while reducing harmful effects. Therefore, the term “probiotic” is derived from the Greek phrase pro bios, meaning “for life”, in contrast to “antibiotic”, which translates to “against life” [1]. The term “probiotic” was first introduced by Lilly and Stillwell in 1965 to describe compounds produced by bacteria that support the growth of other microorganisms [2]. In 1989, Fuller redefined probiotics as “live microbial supplements that enhance the gut balance in host animals” [3]. Describing probiotics in wider terms, these can be monocultures or mixed cultures of live organisms and should support the recovery and maintenance of native gut microbiota, providing benefits to their hosts, whether human or animal. However, a lack of fundamentals essential to uniquely describe the meaning of the claim “probiotic” existed until 2013, when a panel of experts was convened by the International Scientific Association for Probiotics and Prebiotics (ISAPP) to critically reanalyse the previous definitions, as well as the large body of literature, concerning this theme. As a result, probiotics were defined as “live microorganisms that, when administered in adequate amounts, confer a health benefit on the host” [4], a definition that is accepted until nowadays and emphasises their microbial, viable, and health-promoting nature, while differentiating probiotics used for health benefits from those serving as processing aids or sources of beneficial compounds.

Health Canada recognises certain bacterial species as probiotics when provided in food at a cell density of 1 × 10^9^ colony-forming units (CFU) per serving. These include species from the *Bifidobacterium* (*B.*) genus (e.g., *B. adolescentis*, *B. animalis*, *B. bifidum*, *B. breve*, and *B. longum*) and members belonging to the previous *Lactobacillus* (L.) genus, re-classified taxonomically by Zheng et al. [5] in 23 additional genera, (e.g., *L. acidophilus*, *L. gasseri*, *L. johnsonii*, *Lacticaseibacillus* (Lc.) *casei*, *Lc. paracasei*, *Lc. rhamnosus*, *Ligilactobacillus* (Lg.) *salivarius*, *Lactiplantibacillus* (Lp.) *plantarum*, and *Limosilactobacillus* (Ls.) *fermentum*). These species are well-documented for their general health benefits, particularly for maintaining a healthy gut microbiota [6], and serving as fundamentals for permissible claims on Canadian food products.

Similarly, some European Union countries recommend consuming specific probiotic strains for health and nutritional purposes. In Italy, using beneficial bacteria as food supplements or ingredients has been a common practice to support intestinal health. The Italian Ministry of Health has regulated probiotics in the food sector by defining the conditions for the use of the claim, including a daily administration of at least 1 × 10^9^ CFU, full genetic characterisation of the strain, and a demonstratable history of safe use in the market [7].

### 1.2. Beneficial Effects of Probiotics on Human Health

The general benefits of probiotics for gut microbiota stem from their ability to support a more favourable environment in the gut, widely defined as gut homeostasis or eubiosis [8], which is achieved through mechanisms commonly shared by most probiotic strains alongside with dietary regimens. As stated by the ISAPP panel of experts [4], these benefits can be broadly categorised into two main areas: promoting a healthy digestive system [9] and supporting optimal immune system functionality [10].

A substantial body of evidence, including reviews and high-quality meta-analyses examining a wide range of clinical outcomes for various probiotic strains across well-studied species, strongly supports their role in improving digestive health [11,12,13,14,15]. These findings suggest that many strains may exert shared or “core” effects on gut health and physiology.

The immune-boosting benefit of probiotics is also widely acknowledged, but it is often considered more strain-specific [16]. The widespread interpretation of “supporting a healthy immune system”, ranges from allergy prevention [17] to anti-inflammatory effects [18] and enhanced defence against infections [19], which precludes its classification as a significant benefit. Other emerging areas, such as benefits for the reproductive system, oral health, respiratory tract, skin, and the gut–brain axis, show promise but currently lack sufficient evidence across a broad spectrum of probiotics to be considered universal.

**Figure 1:**
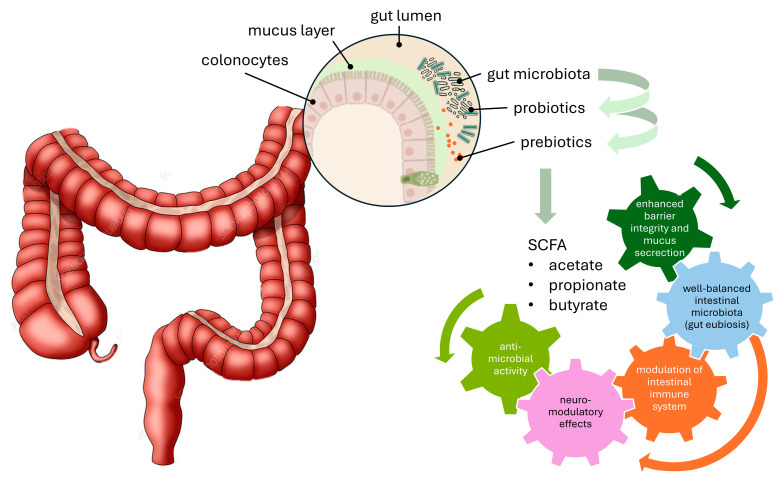
Some mechanisms by which probiotics exert beneficial effects on the host.

Our literature review concerning the mechanisms underlying probiotic effects highlights some that are common across taxonomic groups, such as pathogen inhibition or the production of beneficial enzymes and metabolites [20,21]. However, more specific effects, such as those targeting the immune system or extraintestinal functions, are likely to vary between strains [22]. Health claims related to such effects require mechanistic evidence specific to the strain or species under consideration. However, it is also plausible that a single probiotic strain may exhibit multiple health-promoting actions (Figure 1), such as strengthening the epithelial barrier, competitively excluding pathogens, producing antimicrobial substances, and modulating the immune response [23]. Probiotics impacting toll-like receptors (TLRs), nucleotide oligomerisation domain-like receptors (NLRs), lactose intolerance, diarrhoea prevention (including infectious and antibiotic-associated diarrhoea, *Clostridium difficile*-related diarrhoea, and traveller’s diarrhoea), inflammatory bowel syndrome, urogenital infections, gastric ulcers, food allergies, obesity, cholesterol levels, diabetes, liver diseases, cancer prevention, and oral health (dental caries and orthodontic care) have also been reported.

One notable meta-analysis conducted by Ritchie and Romanuk evaluated a broad range of probiotic strains [24]. This analysis, which encompassed 74 studies, 84 trials, and over 10,000 participants, concluded that probiotics are generally beneficial in treating and preventing gastrointestinal disorders. The European Food Safety Authority (EFSA) has already endorsed the grouping of certain probiotic strains into a functional “class”, as seen with yoghurt cultures like *Lactobacillus delbrueckii* subsp. *bulgaricus* and *Streptococcus salivarius* subsp. *Thermophilus* [25]. These strains were approved for aiding lactose digestion based on the well-understood mechanism of encoding the microbial β-galactosidase enzyme without requiring proof that every strain produces sufficient lactase. Similarly, if a core benefit can be linked to a specific structure or activity, data from any strain exhibiting that property may support health claims. Furthermore, it is reasonable to apply the term “probiotic” to species for which systematic reviews or meta-analyses demonstrate a general health benefit, especially in cases in which no specific health claims are made for the product.

### 1.3. Combining Probiotics with Prebiotics: The Beneficial Effects of Synbiotics

Various approaches have been proposed to modulate gut microbiota composition or activity to achieve gut eubiosis. These include faecal microbiota transplantation (FMT), the use of probiotics and other live microorganisms, and the incorporation of non-digestible dietary components like prebiotics. Initially, prebiotics were defined as “non-digestible (by the host) food ingredients that have a beneficial effect through their selective metabolism in the intestinal tract” [26]. This definition was later revised by the ISAPP panel of experts to “a substrate that is selectively utilised by host microorganisms conferring a health benefit” [27]. The updated definition broadened the scope to include compounds that act as prebiotics in sites other than the gut lumen, removing the requirement for fermentation by intestinal microbiota, previously considered a key criterion for prebiotics [26].

With the growing interest in maximising the health benefits of probiotics and prebiotics, various formulations combining both microorganisms and substrates have been studied and proposed as an alternative for enhancing host well-being. These combinations are referred to as “synbiotics”, a term derived from the Greek prefix “syn-“, meaning “together”, and the suffix “-biotic”, meaning “pertaining to life”.

Given the expanding applications of synbiotics, the ISAPP has recently refined its definition to provide greater clarity. Synbiotics are now defined as “a mixture comprising live microorganisms and substrate(s) selectively utilised by host microorganisms that confers a health benefit on the host” [28]. It should be noted that a deeper understanding of the appropriate utilisation of the term synbiotics should be applied and consists of the difference between complementary and synergistic synbiotics according to evidence of statistical significance reached by the sole co-presence of both probiotics and prebiotics or by the evidence that the beneficial effect precludes the selective utilisation of the prebiotic by the probiotic.

Numerous randomised controlled trials (RCTs) have assessed the potential health benefits of synbiotics in humans, ranging from healthy individuals to those with acute or chronic conditions. Studies have focused on adults with metabolic disorders, such as overweight and obesity [29,30], type 2 diabetes mellitus [31], and non-alcoholic fatty liver disease [32]. Other conditions investigated include irritable bowel syndrome [33], surgical infections [34], chronic kidney disease [35], and atopic dermatitis [36]. These RCTs have been complemented by systematic reviews and meta-analyses, which are widely accepted methods for evaluating the evidence supporting health benefits.

However, evidence of health benefits alone is insufficient to label a formulation of live microorganisms and selectively utilised substrates as a “synbiotic”. Such a designation requires additional evidence of selective utilisation, either by the host’s endogenous microbiota (complementary synbiotics) or by the co-administered probiotic strain (synergistic synbiotics) [28].

### 1.4. Challenges for Probiotic Employment in the Food Chain

**Figure 2:**
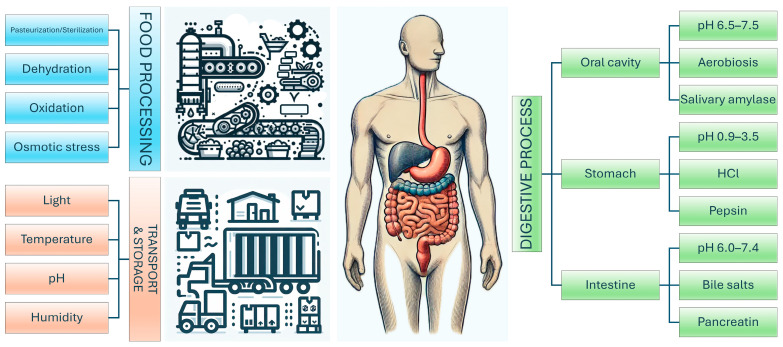
Factors affecting the viability of probiotics.

Employing probiotics in the food chain presents several challenges across various domains, including production, formulation, regulatory compliance, and consumer acceptance. Beyond ensuring the safety of probiotic strains—an essential criterion for the sole application of the claim “probiotic”—one of the primary challenges lies in maintaining the viability and stability of these microorganisms throughout the production process. Probiotics are highly sensitive to adverse conditions, such as heat, oxygen, light, and moisture, which complicates their survival during food processing methods [37]. Additionally, they must remain viable during storage and throughout the product’s shelf life, necessitating strict control of temperature and humidity (Figure 2).

Another critical challenge is ensuring probiotics can survive the acidic environment of the stomach and the presence of bile salts in the intestine to deliver their expected health benefits [38].

Integrating probiotics into food matrices introduces further complexities. Compatibility with different food products is vital, as probiotics may alter the taste, texture, or appearance of the food. For instance, some strains may produce off flavours or interact unfavourably with food components [39,40].

Interactions with other food ingredients also pose challenges. Preservatives and antimicrobial agents commonly used in processed foods [41], as well as polyphenols and organic acids characterising the food matrix, can inhibit probiotic survival and activity [42]. Moreover, naturally occurring microorganisms in fermented foods and the indigenous microbiota of the ingredients may outcompete added probiotics, reducing their effectiveness.

#### 1.4.1. Probiotic Processing: Thermal, Oxidative, and Osmotic Stressor Agents

Probiotics are often subjected to low temperatures during storage, both in bacterial formulations before food production and in refrigerated food products. Additionally, freezing and freeze-drying, which are commonly used techniques for preserving and concentrating probiotics, expose cells to cold stress [43].

Low temperatures during storage can result in cell membrane stiffening, impairing enzyme activity and reducing the rates of RNA transcription and protein translation, which can impair cell growth [44]. Ice crystals formed during freezing can physically damage the bacterial cell envelope by structurally altering it. Furthermore, as water freezes into ice during cold storage, solutes accumulate inside the cells, leading to desiccation and increased osmotic pressure. Since many probiotics are marketed in freeze-dried forms, their ability to survive in cold environments is crucial. For example, *Propionibacterium freudenreichii* was reported to increase branched-chain fatty acid levels in its membrane, derived from branched-chain amino acids, to maintain the required membrane fluidity under cold stress [45].

In lactobacilli, cold stress triggers the production of antifreeze and cold-shock proteins (CSPs), which bind to RNA to prevent the formation of secondary structures [46,47]. This ensures the continuation of transcription, translation, and ribosomal functions, keeping the cells metabolically active under cold conditions. Additionally, certain enzymes secreted by lactic acid bacteria (LAB) exhibit freezing resistance, supporting both RNA and protein synthesis at extremely low temperatures.

Ice crystal damage caused during freezing can also be mitigated by the expression of antifreeze proteins in probiotic LAB strains [48]. To counteract osmotic pressure changes induced by low temperatures, these bacteria secrete and accumulate compatible solutes such as glycerol, trehalose, and amino acids. 

By contrast, heat is a common stress factor in food production technologies that probiotics often face during various stages, such as preparation and processing. Temperatures as high as 60 °C may occur during food preparation, and even brief heat shocks up to 200 °C can result from spray drying processes. High temperatures can denature biomolecules like DNA, RNA, and proteins, disrupting their native properties and hindering metabolic processes. Additionally, heat stress increases cell membrane fluidity, potentially leading to cell damage and death [49]. While bacterial cells can endure mild heat up to 65 °C, it can destabilise non-covalent bonds, disrupt cell envelopes, impair ribosomal function, and denature proteins [50].

Probiotic bacteria often face oxidative stress during food processing and gastrointestinal transit, particularly under aerobic conditions. Oxygen can become toxic by interacting with iron in heme-dependent cytochrome oxidase within the electron transport chain, leading to the formation of reactive oxygen species (ROS). These include superoxide (O_2_^−^), hydroxyl radicals (HO•), and hydrogen peroxide (H_2_O_2_), which are highly reactive and unstable. ROS initiate oxidative chain reactions that damage vital biomolecules such as proteins, DNA, RNA, and lipids, ultimately compromising cell viability [51]. 

ROS are capable of freely crossing the semi-permeable bacterial membrane, and high levels can inhibit microbial replication. Many LABs and bifidobacteria lack enzymes like catalase and superoxide dismutase, which are essential for neutralising hydrogen peroxide and other ROS, making these bacterial strains particularly susceptible to oxidative damage [52].

Osmotic stress affects probiotics when they are exposed to variations in solute concentrations during food processing, such as salt, high sugar content, or brine [53]. This stress increases osmotic pressure, causing water to leave the cells, leading to shrinkage, loss of turgor pressure, and changes in cytoplasmic solute composition, ultimately compromising their viability.

To counteract osmotic stress, probiotics rely on compatible solutes, either absorbed from their environment or synthesised internally [54]. These solutes are often uncharged at a neutral pH, allowing them to accumulate in high concentrations without disrupting cellular metabolism [55].

#### 1.4.2. Storage and Transport 

After production, probiotic products must endure transportation and storage, often lasting over 12 months. During this period, bacterial cells may encounter environmental stressors, including fluctuations in temperature, humidity, oxidative conditions, pH changes, and light exposure [56]. One major concern during storage is the oxidation of membrane lipids, which can compromise cell integrity. For dried probiotic cells, maintaining low relative humidity is crucial to preserve their protective dehydrated state. Overall, lower temperatures and reduced humidity levels are key factors in enhancing the survival of probiotics during storage.

#### 1.4.3. Harsh Conditions in the Gastrointestinal Tract

In addition to enduring the stresses of manufacturing, storage, and transportation, probiotic products must retain their activity and viability as they pass through the human body. Depending on the product type, the dried cells must undergo rehydration either before or during consumption. This process poses a challenge for the cell membrane, as it requires transitioning from a gel-like state to a liquid crystalline state.

Once rehydrated, the bacterial cells must navigate the harsh conditions of the upper gastrointestinal tract (GIT). The stomach is the first major hurdle, where gastric juice exposes the probiotics to an acidic environment (pH < 3) and high concentrations of pepsin, both of which can cause cell damage and death. The severity of this challenge depends on factors such as the pH, composition, buffering capacity, and volume of the gastric juice, as well as the transit time, all of which are influenced by recent food intake [56]; strain-specific variability should also be considered [57,58].

The second significant challenge occurs in the duodenum of the small intestine, where probiotics face bile salts. Bile is more detrimental to bacterial cells than acidic pH because it acts as a detergent, disrupting cell membranes. It can alter cell membrane lipids, affecting permeability and membrane–environment interactions. Bile tolerance varies among strains and is influenced by the production of bile salt hydrolase (BSH) enzymes, which enhance resistance to bile salt exposure [59]. While not all probiotics possess this trait, the ability to hydrolyse bile salts is considered a critical probiotic feature by the World Health Organization [60]. Additional adaptations to bile stress in some LAB strains include active bile salt efflux mechanisms and modifications to the cell wall and membrane composition. Beyond bile salts, the small intestine also contains pancreatin and lipase, which pose further risks to bacterial survival [54]. To exert beneficial health effects, probiotics must remain viable and functional after passing through the digestive system. Key probiotic activities include interactions with the existing microbiota, strengthening the epithelial barrier, immunomodulation, production of antimicrobial compounds, gut colonisation, and biofilm formation [28].

## 2. Probiotics in Functional Foods and Dairy Functional Foods

Food is a fundamental resource and a necessity for meeting the nutritional needs of every human being. In response to the growing awareness among consumers of the importance of food choices, there is an increasing demand for foods that not only meet basic nutritional requirements but also offer additional health benefits. This phenomenon has led to the development and integration of probiotics into several product categories, including nutraceuticals, functional foods and enriched drinks, which are becoming increasingly relevant in the market [61].

Although the idea of “functional foods” has been defined several times, the phrase does not yet have a globally recognised definition. Functional foods are often defined as those that provide additional ingredients that can improve health in addition to necessary nutrients [62]. These advantages might include enhanced immune system performance, better digestion, a lower chance of developing chronic illnesses, and general wellness.

The research, development, and commercialisation of functional foods have increased significantly in the food industry in recent years, focusing on using probiotics to create fortified commercial goods. These meals combine their nutritional value with the addition of probiotics, which are good for the body when taken in sufficient amounts. Most commercially available probiotic-enriched foods are enriched with free probiotic microorganisms, while only a small fraction utilise their microencapsulated form [63]. Table 1 lists some examples of commercially available products enriched with encapsulated probiotics.

The use of free probiotics in functional foods exposes them to numerous stress factors, whereas the use of microencapsulated probiotics ensures that they are better protected and reach their target site to carry out their beneficial functions for the organisms. Nevertheless, adding microencapsulated probiotics to functional foods still presents several difficulties. These include managing production costs, which can be significant given the intricacy of the encapsulation process, choosing the right coating materials, and ensuring that probiotics are distributed throughout the food matrix properly without changing their organoleptic properties.

Dairy foods fortified with probiotics, such as yoghurt, cheese, fermented milk, and ice cream, are among the most developed and studied functional food categories today. Dairy foods are ideal for the addition of probiotics because, as a staple of many people’s diets, they are easily accepted by consumers, who recognise the added health value of these foods. Table 2 shows the different types of probiotic cheeses already studied, detailing species and densities of cultures added and their beneficial contribution.

Despite challenges, the increasing focus on these functional foods has promoted research to overcome these barriers through the development of microencapsulated probiotics, generating numerous scientific studies supporting their benefits. For example, Adhikari et al. [75] investigated the effect of microencapsulation on bifidobacteria in yoghurt, using κ-carrageenan as a coating material. The study showed that this technique improved the survival of *Bifidobacterium longum strains* while preserving the sensory properties of the product. In 2018, Wang et al. [76] applied a similar technique, microencapsulating *Lactobacillus acidophilus LA-5* with polymerised whey proteins, obtaining a significant improvement in the survival of the probiotic and the physicochemical properties of yoghurt, highlighting the effectiveness of microencapsulation in preserving the viability of probiotics in dairy products. Patrignani et al. [77] instead studied the use of high-pressure (HPH) microencapsulation to protect the probiotic lactic acid bacteria *Lacticaseiobacillus paraca-sei A13 and Ligilactobacillus salivarius* subsp. *salivarius CET 4063* to produce functional fermented milk. The bacteria were microencapsulated through an HPH treatment using a solution of sodium alginate and vegetable oil. The results showed that this technique not only improves the protection of probiotics but also the sensory properties of fermented milk, suggesting new possibilities for the development of innovative functional foods. The microencapsulation technique was also subsequently evaluated in a study conducted by Kavas et al. [78] to preserve the viability of probiotic bacteria *Lacticaseibacillus paracasei* and *Bifidobacterium longum* in white goat’s milk cheese during storage. In particular, different types of microcapsules were used, some containing only probiotics and prebiotics, such as fructooligosaccharides (FOS) and inulin, and others with only probiotics or prebiotics. The results of the study showed that microencapsulation preserved the viability of probiotics, with better performance in cheeses enriched with prebiotics and that cheeses containing both probiotics and prebiotics maintained bacterial levels above the therapeutic minimum threshold, indicating that they can be considered effective carriers of probiotics and offer potential health benefits. 

### Probiotic Fortified Pasta Filata

The development of foods that maintain an adequate amount of probiotics at the time of consumption is a complex challenge, as various factors related to processing and storage, such as pH, temperature, oxygen, and the presence of antagonistic microorganisms, can compromise probiotic viability [79]. Although numerous studies have investigated the development of probiotic cheeses [80], few have focused on the incorporation of probiotic bacteria (such as *Lactobacillus* or *Bifidobacterium* spp.) into pasta filata cheeses, such as mozzarella. Therefore, this review focuses mainly on pasta filata cheeses enriched with probiotic microorganisms. Pasta filata cheeses comprise several varieties made from cow, buffalo, goat, or sheep milk. They involve a production process in which the curd is stretched in hot water, which gives these cheeses a soft or semi-soft consistency; they can be eaten fresh or after a short ripening period. Well-known examples include mozzarella and fiordilatte, as well as scamorza and burrata [81].

Numerous challenges are associated with adding probiotics to pasta filata cheeses, the most significant being the survival of these bacteria at the water’s high temperature during stretching and storage. Another challenge found when adding a probiotic to a food is maintaining the sensory attributes of the product. In addition, it is essential to ensure that the cheese maintains a sufficient probiotic load throughout its shelf life to guarantee health benefits. Pasta filata cheeses often have a limited shelf life, which makes it difficult to maintain a high concentration of probiotics throughout the life of the product [82]. In response to these limitations, various researchers have explored methods to enhance probiotic survival in pasta filata cheeses. For instance, Minervini et al. [83] focused on selecting thermoresistant probiotic lactobacilli to produce fiordilatte cheese. After evaluating the resistance of 18 strains of lactobacilli, *Lactobacillus delbrueckii* ssp. *bulgaricus SP5* and *Lacticaseibacillus paracasei BGP1* were selected, as they showed greater survival in sublethal heat adaptation treatments that simulated curd stretching. The results demonstrated that the tested strains reached a high cell density, particularly exceeding 8.0 log10 CFU/g during 15 days of storage at 4 °C, despite an initial decrease in probiotic cell density due to the stretching phase at 80 °C (core temperature of the curd at 55 °C). Similarly, the study by Cuffia et al. [84] conducted in 2017 experimented with the adaptation of specific technological parameters, such as curd acidification (pH 5.25), time (2, 5, 10, and 20 min), and stretching temperatures (58, 62.5, and 68 °C), to obtain a pasta filata cheese capable of maintaining probiotic bacterial levels above 10^7^ CFU/g. Among the five probiotic strains tested, *Lacticaseibacillus rhamnosus GG* was the most heat-resistant and was added to pasteurised milk at a final concentration of 5 × 10^7^ CFU/mL. A stretching temperature of 62.5 °C maintained for 10 min resulted in probiotic viability reduction of 0.44 log10 CFU/g. However, this treatment still ensured that probiotic levels remained above 7.5 log10 CFU/g during a 15-day storage period at 4 °C. Both studies highlighted that adjusting technological variables and using sublethal stress can improve probiotic viability during storage at 4 °C in fiordilatte cheese, supporting its potential as a probiotic carrier. A subsequent study by Akarca et al. [85] evaluated the effect of probiotic bacteria on different aspects of mozzarella, such as chemical parameters, texture, and sensory characteristics, using cow’s milk and buffalo milk. The results showed that, during the storage period, the *Lactobacillus acidophilus* count increased significantly despite an initial reduction in the number of probiotics inoculated due to scalding during the production phase. However, with the extension of storage, the increase in *Lactobacillus acidophilus* was similar in both types of milk, with no significant differences attributable to the type of milk used. In the same way, a study by Reale et al. [79], which aimed at the production of two probiotic Italian pasta filata cheeses by direct inoculation of probiotics in vats, highlighted that the curd stretching phase severely compromises the viability of probiotics, reducing the probiotic load by more than 2 log CFU/g. Consequently, in this study, mozzarella did not prove to be a good vehicle for probiotics since the period of storage at refrigeration temperature did not allow for the restoration of a sufficient quantity of probiotics. On the contrary, it was observed that maturation of at least 30 days at 15 °C favoured an increase in the probiotic load from 10 to 100 times. This demonstrates that semi-mature cheeses, such as scamorza, which are obtained via direct inoculation in the vat, can represent an effective food vehicle for probiotics. In 2013, instead, a study by Albenzio et al. [86] investigated the effect of probiotics on the composition and sensory properties of scamorza produced with sheep’s milk. After the thermal adaptation of the bacterial strains at 65 °C for 30 min, *Lactobacillus acidophilus* LA-5 and a mixture of *Bifidobacterium longum* BL-46 and *Bifidobacterium lactis* BB-12 were inoculated into milk. Cheeses named S-BB (with *Bifidobacterium* spp.), S-LA (with *Lactobacillus acidophilus*), and a control (S-CO) were produced. After 15 days of ripening, the probiotic cheeses maintained high bacterial viability (7.55–9.9 log CFU/g) and presented better sensory characteristics than the control, confirming the potential of scamorza as a probiotic functional food. In a subsequent study in 2023, Alsaleem et al. [87] investigated the effect of the addition of skimmed milk powder (SMP) and whey protein concentrate (WPC) on the survival of probiotics in mozzarella. Five variants of probiotic mozzarella were produced: a control sample (PMC1) with probiotics only and four others with probiotics and varying concentrations of SMP and WPC. During the 28 days of storage, the control sample recorded the lowest probiotic bacteria (BB) count, decreasing from 6.45 CFU/g to 5.42 CFU/g. However, the addition of WPC showed a positive effect on the stability of probiotics, maintaining the BB count above 6 log CFU/g in samples PMC4 and PMC5. The addition of skimmed milk powder also contributed to the survival of bacteria, but to a lesser extent than WPC. These studies highlight how storage time plays a crucial role in the stability of probiotics and how the addition of specific additives can promote their survival in mozzarella cheese. 

Another approach to enhancing probiotics in dairy products was explored by Ortakci et al. [88] and Mukhtar et al., both of whom analysed the effectiveness of encapsulation in alginate systems to improve the survival of specific *Lactobacillus* strains during the production and storage of mozzarella. Mukhtar et al. [89] focused on the strain *Lactobacillus acidophilus S2*, demonstrating its resistance to high temperatures (55–65 °C) and acidic conditions (2–3). Cheeses containing free and encapsulated cells were compared, showing that encapsulation significantly improved probiotic viability. Similarly, Ortakci et al. studied the survival of *Lacticaseibacillus paracasei LBC-1* in partially skimmed mozzarella, observing that during production, in which the curd is heated to 55 °C and then immersed in brine at 70 °C, encapsulated probiotics showed greater resistance than free cells, with a vitality reduction of 0.25 log compared to a reduction of 0.45 log for non-encapsulated bacteria. Both studies showed how encapsulation is an effective strategy to protect probiotics, contributing to their stability and functionality during processing and storage. 

Alginate was also used in a study conducted by Angiolillo et al. [80], in which an innovative method for producing a synbiotic mozzarella cheese was proposed. The innovation lies in the application of an alginate coating on the surface of the product by immersing the latter, which is used as a carrier of probiotic and prebiotic substances. In particular, the edible coating was enriched with *Lacticaseibacillus rhamnosus* and FOS via direct incorporation into the solution. This approach was shown to be effective in supporting the viability of probiotics during storage at different temperatures, particularly at 4 °C, 9 °C, and 14 °C. These are some examples of probiotic fortification in pasta filata cheeses, particularly fiordilatte, mozzarella and scamorza, which demonstrate the strong potential of these cheeses as effective probiotic carriers. 

The addition of probiotic microorganisms in pasta filata cheeses is used not only to produce fortified pasta filata cheeses but also to delay the deterioration of the product quality and therefore prolong its shelf life. Burrata, for example, is a fresh pasta filata cheese produced in Italy whose demand on the world market is continuously growing; however, due to its naturally poor competitive microbiota, relatively high values of water activity and slightly acidic pH, various microorganisms, especially bacteria, can grow in burrata, thus causing various deteriorations that limit shelf life. One of the approaches used to prevent the growth of undesirable microorganisms and prolong the shelf life of fresh cheese is the application of bioprotective cultures that do not negatively affect the sensory quality of foods. This approach was used in a 2017 study by Minervini et al., who employed the protective probiotics *Lactiplantibacillus plantarum LPAL* and *Lacticaseibacillus rhamnosus LRB* to inhibit undesirable bacteria with or without the addition of FOS and inulin in burrata cheese. Overall, the addition of inulin and FOS and the use of protective strains modified the microbiome assembly of burrata cheese, and it was shown that protective lactobacilli guided microbial communities, as well as reducing *Streptococcus* and *Lactococcus* contamination during cheese production, and slowed the growth of staphylococci, coliforms, and *Pseudomonas* spp., especially in early storage. Thus, the bacterial groups that were inhibited are all recognised as pathogens/spoilage bacteria of fresh cheeses that reduce the shelf life of the products. Therefore, this study demonstrated the effectiveness of an innovative biotechnology that combines the use of prebiotic dietary fibres and protective probiotics [90]. This approach not only inhibits the proliferation of pathogenic and spoilage bacteria in fresh cheeses, thereby extending their shelf life, but also offers the opportunity to develop fortified products capable of acting as vehicles for probiotics. Further in vivo analyses could confirm and enhance the benefits of this strategy, thus paving the way for new applications in the functional food sector.

## 3. Microencapsulation of Probiotics: A Strategy to Increase Probiotic Vitality and Overcome Challenges

In recent years, the global market for probiotic-based functional foods has expanded significantly, largely due to increased awareness of gut health’s crucial role in overall well-being and the essential functions of the microbiota. This heightened awareness has increased demand for probiotic products, driving industry growth and research. For probiotics to be effective, enough live and functional organisms (6 log CFU/g) must be present both in the product and at the site of action [91]. Despite considerable scientific advancements, several challenges remain in ensuring that probiotics can fully express their therapeutic potential due to their high sensitivity to the numerous stressors they encounter before reaching their site of action.

The reduced viability of probiotics poses a considerable challenge to the effectiveness of commercial products currently on the market. Many probiotic products may not be as effective as claimed [58]. Research has shown a marked decline in the concentrations of these products compared to their reported claims, highlighting issues with probiotic strain viability as they move through the GIT [92]. These findings emphasise the need for innovative strategies to improve the survival and viability of probiotics throughout the entire process, from production to administration. Advances in encapsulation technology are crucial for protecting microorganisms during their passage through the GIT, releasing them only once they reach the intestinal environment. Microencapsulation has emerged as a prominent technique, offering a novel approach to tackling stability and survival issues associated with probiotics [93]. This process involves encapsulating probiotic strains in a polymer matrix, creating a protective layer that regulates their release. Microencapsulation is an advanced technique that involves coating microscopic particles or droplets of active substances with a protective material, forming microcapsules that range from 1 to 1000 µm in size [94]. This technique provides a physical barrier to probiotic cells, safeguarding them from stress, preventing cell injury, and enhancing their survival and viability, thereby improving their health benefits [95,96]. Microencapsulation enables the controlled or targeted release of probiotics, optimising their bioavailability, functionality, and efficacy. Another benefit is that it can mask the distinctive taste and aroma of probiotics, which might otherwise affect the acceptability of food and beverage products. Encapsulation helps conceal these sensory characteristics, enhancing the palatability and acceptance of products [97]. Furthermore, microencapsulated probiotics can be incorporated into a wide range of formulations, including foods, beverages, and supplements. The extended shelf life of probiotics due to microencapsulation is also significant, as it helps maintain their viability and efficacy over time, ensuring they remain active until consumption.

The choice of material to be used in the probiotic microencapsulation process is crucial, as it must ensure not only physical protection of probiotic cells but also a controlled and targeted release at the site of action. The materials used for microencapsulation must be food-grade, biodegradable, and able to form a physical barrier against numerous stress factors. Furthermore, they must be compatible with the probiotics and possess specific chemical–physical properties, such as solubility in water, film-forming capacity and adequate molecular weight [98].

Among the most used natural materials are polysaccharides, such as alginate, carrageenan, starch, cellulose, gum arabic, pectin and chitosan, which offer excellent protective properties and good stability. For example, alginate is one of the most widely used materials due to its ability to form strong, stable gels in the presence of divalent cations such as calcium. Typical concentrations of 1–3% (weight/volume) create an effective barrier that protects probiotics from the acidic pH of the stomach, allowing them to be released into the intestinal tract. However, alginate can have limitations, such as high porosity and low resistance to extreme acidic conditions, which can be improved by combining it with other polymers. Proteins, such as whey, casein and soy proteins, are another widely used class of materials for microencapsulation. These offer good protection to probiotic cells and can be combined with polysaccharides to improve the stability of microcapsules [99]. These materials are often combined to exploit the unique properties of each, enhancing the protection and release of probiotics. In addition to choosing the most suitable polymer, it is important to evaluate the ratio of polymeric material to probiotics. The amount of encapsulation material must be adequate to protect the probiotics during the GIT but without compromising their viability or release at the site of action. The ideal ratio varies depending on the material used and the encapsulation technique. A balanced ratio ensures that cells are protected but not excessively encapsulated, ensuring that a minimum amount of probiotics (6 log CFU/g) reaches the site of colonisation [100].

## 4. Techniques for Microencapsulation of Probiotics

Various microencapsulation techniques are available, including extrusion, emulsion, fluidised bed, freeze-drying, spray-freezing, spray chilling, electrospraying, and microfluidic. Choosing the optimal microencapsulation technique requires a thorough assessment of the probiotics to be encapsulated, the intended application, product requirements, and a cost–benefit analysis. Importantly, the chosen technique must not be harmful to the cells. The following section will discuss the most popular probiotic encapsulation technologies.

### 4.1. Nozzle Extrusion Techniques (Prilling/Vibration Technique)

**Figure 3:**
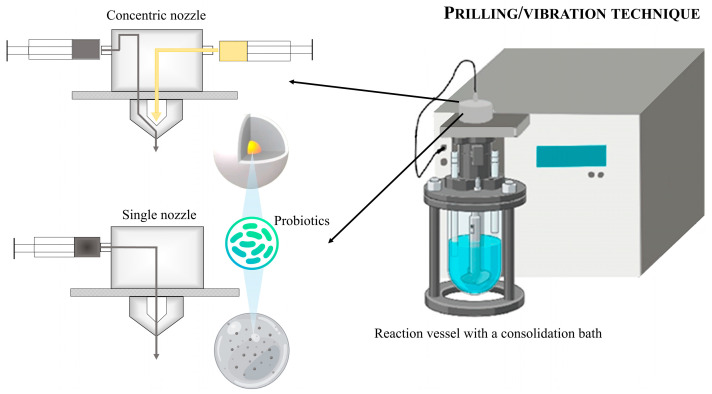
Illustration of probiotic encapsulation using the prilling/vibration technique. Figure licensed under a Creative Commons CC-BY 4.0 license; adapted with permission from [105].

The extrusion technique is a physical method based on mixing the probiotic cells with the polymer solution, followed by extrusion into a cross-linking solution through a needle or syringe nozzle [101]. There are several technologies to produce microparticles by extruding a liquid through a nozzle or orifice, which are based on different mechanisms for breaking up the liquid jet: conventional dripping (simple extrusion), electrostatic cutting, a jet cutter or mechanical cutting technology with a rotating disc or jet cutting, and a vibrating nozzle [102]. Prilling/vibration is an innovative technique based on breaking a laminar jet of polymer solution into one-dimensional droplets using a vibrating nozzle device. To prevent the droplets from coalescing, an electrostatic charge is induced on their surface, which then falls into a consolidation bath, where they solidify into microparticles. The system can produce both microcapsules and microspheres with a very narrow size range (Figure 3) [103,104].

The prilling/vibration method is a technique that does not require high temperatures or solvents, ensuring high cell viability. This encapsulation process is efficient, rapid, reproducible, and industrially scalable [106,107]. It also allows the use of natural polymers such as alginates, pectin, carrageenans or gelatins, which are biocompatible and safe. *Lactobacillus acidophilus* and a mixture of *Bifidobacterium longum* and *Bifidobacterium lactis* were microencapsulated in alginate microparticles to be added to lamb rennet paste to protect the cells during cheese production [108]. In another study [109], probiotic lactic acid bacteria were microencapsulated with whey–alginate–pectin or whey permeate–alginate–pectin, and the microparticles protected the bacteria from simulated gastrointestinal conditions and during storage for 3 months at 4 °C. Furthermore, the encapsulation slowed down the acidification process of the milk, making these particles suitable for incorporation into food without altering its sensory properties, such as taste and texture. The combination of several polymers allows the system to be customised to ensure a specific delivery. For instance, in the work by D’Amico et al. [110], multi-stimuli responsive microcapsules for targeted colonic delivery of *Lactobacillus plantarum* 4S6R were produced via the prilling/vibration technique. Microcapsules provided significantly higher viability of microencapsulated probiotics than free probiotics in simulated gastric intestinal media with bile salts and were able to protect probiotics when they were subjected to thermal stress.

Despite the many advantages of the prilling/vibration technique, there are some limitations to consider, which present a challenge for researchers to overcome. For example, the size of the microparticles produced is influenced by several factors, including the size of the nozzle, the viscosity of the polymer solution, and the distance between the syringe and the cross-linking solution; thus, small variations can significantly affect the size distribution. The technique allows for the use of a significantly reduced range of polymers, as these must be able to crosslink in a suitable consolidation bath [101] and require a subsequent drying step.

In the literature, several works analyse the microencapsulation of probiotics using extrusion techniques for insertion into pasta filata and cheese; examples are shown in Table 3.

### 4.2. Emulsion Technique

The emulsion technique is another widely used approach for the microencapsulation of probiotics. In general, emulsion is a colloidal dispersion formed by two or more immiscible liquids (usually water and oil). Emulsion systems can be divided into traditional emulsion systems and multiple emulsion systems according to the number of different phases they contain. In traditional emulsion, a small volume of the cell–polymer suspension (discontinuous phase) is added to a large volume of the continuous phase and, according to the relative composition of the two phases, is classified as water-in-oil (W/O) and oil-in-water (O/W) emulsion [111,112]. Multiple emulsion systems consist of emulsified emulsions, such as water-in-oil-in-water emulsions (W_1_/O/W_2_) or oil-in-water-in-oil emulsions (O_1_/W/O_2_). Due to the surface hydrophilicity of most probiotic cells, W_1_/O/W_2_ emulsions are most commonly used to encapsulate probiotics because the probiotics can be trapped within the inner aqueous phase and thus protected from environmental stresses. Once the emulsion is formed, it can be added directly to dairy products such as yoghurt, cheese, or pasta filata, or if a polymer is present in the discontinuous phase, it can be insolubilised (cross-linked) to form tiny gel particles within the continuous phase. A reduction in the particle size of the internal emulsion phase will result in a corresponding reduction in the size of the final microparticles. The insolubilisation method to be chosen depends on the type of polymer used [112] and can be either internal or external. In the case of alginate, internal gelation is induced by adding an oil-soluble organic acid, such as acetic acid, to the emulsified mixture. The organic acid reacts with the calcium carbonate in the alginate, releasing calcium ions that interact with the carboxyl groups in the alginate, forming the typical egg-box structure [113]. In other cases, gelling can occur externally by immersing the emulsion droplets in a gelling solution. The solidified microcapsules are separated from the reaction mixture via filtration or centrifugation and washed to remove any residual oil, emulsifier, or gelling solution.

The emulsion technique traps or immobilises probiotic cells within microcapsules or microparticles, which provide protection from environmental stresses and ensure the survival of the microorganisms. This type of system can increase the viability of probiotics under gastric conditions because the oily phase protects the probiotics from acidic gastric fluids; it is also an easy technique to scale up industrially, and the resulting capsules have small diameters [114]. Moreover, the emulsion technique operates under relatively mild conditions, which is crucial to preserving the viability of heat-sensitive probiotic bacteria [115]. However, the main disadvantages of this method are the instability, coalescence of the emulsion droplets, and inhomogeneity in size and shape. Furthermore, the emulsion technique with interfacial polymerisation presents some critical issues related to the difficulty of removing the organic solvent. In fact, in practice, halogenated organic esters such as methyl chloroacetate and ethyl chloroacetate are chosen as dispersing solvents [116].

Due to its interesting technical and economic advantages, probiotic encapsulation via the emulsion method and its application in fermented dairy products are rapidly expanding the area of research [88], and examples are reported in Table 3. Therefore, these studies highlight how the emulsion encapsulation technique represents a promising solution to overcome the challenges associated with the survival and functionality of probiotics in dairy products. Applying this technique not only improves the stability of the bacteria during production processes but also ensures that their therapeutic efficacy is maintained throughout the product life cycle, offering new opportunities for innovations in the food industry.

**Table 3:** Examples of microencapsulated probiotics produced using different techniques applied in dairy functional foods.

Type of Cheese	Microencapsulation Technique	Probiotic Strain	Encapsulating Material	Main Results	Reference
Kariesh cheese	Prilling/vibration technique	*Bifidobacterium lactis* BB-12, *Lacticaseibacillus rhamnosus* NRRL B-442 and *Lactobacillus gasseri* NRRL B-14168	Sodium alginate and rice flour	The survival rate of probiotics exposed to in vitro simulated GI solutions was recorded at 72.9	[117]
White soft cheese	Prilling/vibration technique	*Bifidobacterium lactis* BB12	Sodium alginate, fish oil, and pomegranate peel extract (PPE)	The probiotic + fish oil + PPE emulsion protected the probiotic bacteria during storage for 30 days	[118]
Goat Ricotta	Prilling/vibration technique	*Lactobacillus acidophilus* (La-05)	Alginate and chitosan	Microencapsulation of probiotic cultures resulted in increased probiotic survival	[119]
Oaxaca cheese	Emulsion	*Lactobacillus plantarum*	Aguamiel/Canola oil/Sweet whey	The inclusion of bacteria in double emulsions provides a physical barrier against deleterious environmental factors	[120]
Chami Cheese	Emulsion	*Lactobacillus plantarum* 564	Camel milk protein and wheat starch	The emulsion technique improves the stability, preservation, and survival of the GI passage of probiotic cells	[67]

### 4.3. Fluid Bed Coating

Fluid bed coating is a widely employed method for the microencapsulation of probiotics, primarily utilised to enhance their stability, protect them from environmental stressors, and allow for their controlled release [121]. It consists of coating solid particles in a suspension with an atomised encapsulating agent. To apply this technique to probiotics, the cells must first be transformed into solid particles, meaning that fluidised bed coating is considered a co-encapsulation technique. In this technique, a probiotic powder or suspension is suspended in a flow of heated air in a fluidised bed, creating a fluid-like state. A coating material is sprayed onto the particles while in motion. The coating material is typically dissolved or suspended in a solvent that evaporates on contact with the heated particles, forming a solid protective layer around the probiotics.

The fluidised bed coating technique is fast, economical, scalable, and versatile, allowing for the use of different materials to customise the coating according to the probiotic strain and the desired product. The primary function of the coating is to protect probiotics from the external environment, including moisture, oxygen, and extreme pH conditions, while also improving their stability and ensuring their viability during storage and transit [122]. For example, Sánchez-Portilla et al. [123] proved that the viability of Bifidobacterium sp. was retained for more than 2 years, with a concentration exceeding 5 log CFU/g, as well as resistance to acid and complete enteric-targeted release, through the fluidised bed drying technique. Fluid bed coating facilitates the incorporation of probiotics into a wide range of products, from dairy to functional foods, without compromising their bioactivity. The technique also allows for the controlled release of probiotics, ensuring their delivery to the intestines, where they can exert their beneficial effects [124]. However, there are also some limitations associated with fluid bed coating. It is a complex technique that requires pre-encapsulation, as the probiotics must be encapsulated and dried before coating in the fluidised bed dryer. One challenge is the potential for uneven coating thickness, which can affect the encapsulation efficiency and the uniformity of probiotic protection [125]. Achieving an optimal coating layer requires careful control of process parameters, such as the spray rate, temperature, and airflow. Additionally, the use of certain coating materials may limit the stability of probiotics, as some materials may not provide adequate protection against harsh GI conditions [126]. Another disadvantage is that the technique may not be suitable for encapsulating high-moisture probiotic suspensions, as the drying step required in the fluid bed process could lead to moisture loss, negatively affecting the probiotic viability [127]. Finally, the cost of coating materials and the need for specialised equipment may increase the overall production cost compared to other microencapsulation techniques, such as spray drying.

Fluidised bed coating has been used to encapsulate probiotics for various foods and to preserve the viability of probiotics in food matrices. Galvão et al. [128] dried and coated apple cubes with a mixture containing hydroxyethyl cellulose and polyethylene glycol containing *B. coagulans* using the fluidised bed drying technique. The viability of the probiotics in the dried apple snacks was well preserved during the storage period. The fluidised bed drying technique was also used by Mirzamani et al. [129] to develop probiotic bread. The double-layer microcapsules produced by the fluidised bed drying technique had a higher heat resistance and could protect the encapsulated probiotics (*L. Sporogenes*) under cooking conditions. In another study [130], carrot tablets containing *L. plantarum* TISTR 2075 were produced using a fluidised bed drying technique with gelatin and showed greater tolerance to thermal digestion treatments than free cells. The fluidised bed drying technique was also used to microencapsulate *Lactobacillus acidophilus* LA-5 and *Bifidobacterium* BB-12 with hydrolysed whey protein and xanthan gum to make functional beverages ready for reconstitution [131].

In conclusion, fluidised bed coating is a versatile microencapsulation technique suitable for large-scale production, which can be used to encapsulate probiotics effectively. However, its complexity and associated costs require careful planning and process optimisation to ensure the highest quality of the final product.

### 4.4. Freeze-Drying

Freeze-drying, also known as lyophilisation, is widely recognised as an effective method for microencapsulating probiotics. This technique plays a crucial role in enhancing probiotic stability, protecting their viability during processing and storage, and ensuring efficient delivery to the GIT [132]. The process involves three primary stages: freezing the probiotic suspension, primary drying (sublimation), and secondary drying to remove residual moisture [133]. Probiotic cells are commonly mixed with cryoprotectants, such as trehalose, sucrose, skim milk, or polysaccharides like maltodextrin, which mitigate cellular damage during ice crystal formation and dehydration [134]. This protective matrix not only maintains cellular integrity but also serves as a barrier against environmental stressors [135].

Studies have shown that *Lactobacillus rhamnosus* GG, when encapsulated with FOS and freeze-dried, exhibited over 90% survival after exposure to gastric acid (pH 2) and bile salts [136]. Similarly, *Bifidobacterium bifidum* freeze-dried with inulin demonstrated superior resistance to storage at room temperature for 12 months compared to non-encapsulated cells [137]. The low operational temperatures of the freeze-drying process also ensure minimal denaturation of sensitive probiotic enzymes and proteins, maintaining their bioactivity during and after reconstitution [138].

Despite its numerous benefits, freeze-drying is not without challenges. The process is energy-intensive, requiring long cycle times, high cost, and specialised equipment, which can limit scalability for industrial applications [139]. Furthermore, inadequate formulation of the cryoprotectant matrix may result in insufficient protection, leading to reduced cell viability under extreme storage or processing conditions [134].

Recent innovations aim to address these limitations by integrating freeze-drying with advanced encapsulation techniques. For instance, the co-encapsulation of probiotics with prebiotics (e.g., FOS) has been reported to synergistically enhance probiotic viability and promote their metabolic activity upon delivery to the host gut microbiota [136,140]. With continued advancements in formulation science and process engineering, freeze-drying is poised to remain an indispensable tool for the development of next-generation probiotic foods and supplements.

### 4.5. Spray Drying

**Figure 4:**
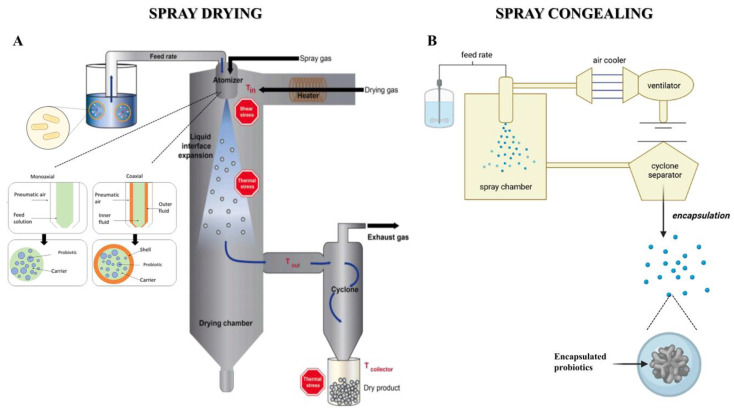
Schematic illustration of spray drying (**A**) and spray congealing (**B**) techniques. Figures licensed under a Creative Commons CC-BY 4.0 license; adapted with permission from [178,179].

Microencapsulation by spray drying is a process used in the food industry to protect probiotics during food processing, storage and passage through the GIT [141]. This method involves several steps, as shown in Figure 4A. First, the polymer solution is prepared, which contains probiotics and a coating material, also known as encapsulating agent or wall material, such as proteins, starches, polysaccharides, or sugars. The mixture is properly homogenised to ensure an even distribution of the components and is then sprayed into a drying chamber through a nozzle, which creates tiny droplets. The atomised droplets come into contact with a stream of hot air or gas, where rapid evaporation of the solvent occurs, resulting in the formation of dry particles of encapsulated probiotics. They are separated from the drying gas by a cyclone that deposits them in a glass collection container [142]. Spray drying is characterised by two distinct configurations, as shown in Figure 4, with two- or three-fluid nozzles that allow for the creation of both matrix microparticles and core-shell microcapsules, respectively.

Spray drying has numerous advantages for the microencapsulation of probiotics, making it a suitable technique for the large-scale production of enriched foods. It is a rapid, continuous, reproducible process that can produce large quantities of microparticles in a short period [143]. The operational cost is low compared to other microencapsulation techniques; in fact, this technique is about 10 times cheaper than freeze-drying [144]. Spray drying is an efficient method, guaranteeing a high production yield and minimising losses during the process. However, the survival of probiotics during spray drying and the resulting encapsulation efficiency are critical parameters that depend on several factors, such as the species and strain of probiotic, inlet and outlet temperatures, atomisation rate, osmotic stress, dehydration stress, and polymers used [145]. As it is a rapid process, however, it is also suitable for temperature-sensitive compounds. Microencapsulation by spray drying improves the stability and shelf life of the probiotics in the product due to the low water activity of the microparticles. Microencapsulation protects the probiotics from harsh conditions during food processing, such as oxygen, light, humidity and those in the GIT, preserving their viability during processing and storage [116]. Nevertheless, spray drying also presents certain disadvantages and challenges, including the necessity of operating under difficult process conditions. High temperatures and osmotic stress can have a negative impact on the viability of probiotics, which are particularly sensitive to heat. Inlet temperatures above 60 °C can damage cells, causing a significant loss of viability, including through damage to the cell membrane and loss of intracellular substances [146]. The inactivation of bacteria due to thermal, osmotic, and oxidative stress during the microencapsulation process is the main challenge that can be overcome by employing various strategies, such as selecting probiotic strains that are more resistant to thermal stress, optimising the drying parameters, and adding thermal and/or osmoprotective agents. Adequate concentration of the coating material is essential to protect the probiotics during the process; however, too high a concentration can slow down evaporation, prolonging the residence time in the drying chamber and increasing the risk of loss of viability [147]. The microencapsulation of probiotics by spray drying is an advanced and valuable technique in the food industry, offering significant advantages for the integration of beneficial microorganisms into a wide range of probiotic-enriched functional foods while preserving their efficacy and stability. Furthermore, probiotic powders obtained via spray drying are ideal for food supplements, as they are stable and easy to dose, facilitating the consumption of probiotics, even outside meals.

In bakery products, this technology allows for probiotics to be incorporated without high temperatures compromising their viability, thus expanding the possibility of creating innovative probiotic foods, such as breads, biscuits, and health snacks [148,149]. For example, Malmo et al. [150] prepared a potentially probiotic chocolate soufflé using *Lactobacillus reuteri* DSM 17,938 cells, which were microencapsulated via spray drying in an alginate matrix and further coated with chitosan. Microencapsulation led to a survival rate of 10% after baking the chocolate soufflé.

The use of different combinations of wall materials for microencapsulation via spray drying *of Lactobacillus casei* has proven to be effective in enhancing the viability of probiotics in a fermented milk dessert [151,152,153]. Microencapsulated probiotics can be added to milk powder to create a functional product with a longer shelf life [154,155,156,157].

The microencapsulation of probiotics in yoghurt has been extensively studied to improve their viability during the storage period, ensuring that the microorganisms remain active for extended periods, even under refrigerated conditions. For example, the incorporation of microencapsulated *Lactobacillus acidophilus* LA-5 via spray drying into yoghurt, using whey powder and gum arabic as wall materials, improved the survival of probiotics during storage and simulated digestion [158]. In another study, microencapsulation via spray drying *Lactococcus lactis* Gh1 in yoghurt with Synspealum *dulcificum* and gum arabic showed high encapsulation efficiency and better viability during storage than non-encapsulated cells [159]. Picot and Lacroix [160] successfully encapsulated Bifidobacterium strains, which demonstrated a high survival rate during spray drying and improved viability of the microencapsulated probiotics compared to free probiotics during 28 days of storage in low-pH yoghurt and a simulated gastrointestinal environment.

The incorporation of microencapsulated probiotics via spray drying into cheeses, such as pasta filata and mozzarella, can improve their nutritional and functional value. These probiotics can maintain their viability during processing, storage, and passage through the GIT and can contribute to the development of desirable flavours and aromas during cheese ripening, as well as improving digestibility and nutrient absorption [64,161]. Examples of dairy functional foods containing microencapsulated probiotics produced via spray drying are shown in Table 4.

Spray drying microencapsulation thus represents a promising technology for the incorporation of probiotics into a wide variety of food products. This method offers numerous advantages, although further research in this field is needed to facilitate the development of more effective probiotic delivery systems and expand the application of this technology into a wider range of innovative functional food products with high nutritional value and health benefits.

### 4.6. Spray Congealing

Spray congealing technology, also known as spray cooling or spray chilling technology, has been widely studied and used in the pharmaceutical and food fields [165]. As shown in Figure 4B, it involves preparing a dispersed solution or emulsion containing the active ingredient and a molten carrier, which is then atomised by a heated atomising nozzle to maintain the correct temperature and avoid the recrystallisation of the substances. When the droplets come into contact with a cooled environment (injection of cold air or liquid nitrogen), there is a heat transfer that leads to the solidification of the matrix and results in spherical particles. The particles are collected in a container below the cooling chamber, while very fine particles are transported by air to a cyclone and collected in another container [166,167,168,169].

The spray cooling method has recently been used as a new technique in food research due to its high protective effect in microencapsulation [170]. Indeed, this technology could generate smaller microspheres, which could be desirable in food processing [171]. The resulting microparticles could protect probiotic cultures from adverse conditions during processing, storage, or the simulated gastrointestinal environment [172]. Other advantages include high encapsulation efficiency, the absence of high temperatures and organic solvents, which are necessary for other techniques but can be toxic/lethal to probiotic strains, compromising not only their viability but also their functionality [169]. Another good aspect of the spray chilling technique is related to the release mechanism of the active ingredient. If the matrix is composed of fats, the release of the bacteria occurs directly in the intestine because of the action of lipases present in the intestinal lumen [165]. Furthermore, spray chilling is considered the most economical encapsulation technology, as it consumes less time and energy than other particle formation methodologies and can be produced on an industrial scale with continuous production [171,173,174]. Microparticles that are produced may have some disadvantages, which include the expulsion of the encapsulated material during storage due to the crystalline structure and polymorphic arrangement characteristic of many lipid materials during the solidification and crystallisation process [171]. Furthermore, the encapsulation of probiotics requires temperatures suitable for the selected strain, as high carrier melting temperatures or low consolidation temperatures may result in the loss of viability of the microorganism.

In the literature, there are a few works in which microencapsulated probiotics have been produced with the spray chilling technique [171,175,176]. In the study by Silva et al. [177], a culture of *Lactobacillus acidophilus* (free or microencapsulated) was added to the requeijão cremoso processed cheese. This study aimed to evaluate the effect of adding probiotic cultures (free or microencapsulated) on the quality parameters of the cheese. Microencapsulation was performed using spray chilling and cottonseed vegetable fat as an encapsulating agent. The microencapsulated probiotic cultures were added before curd-melting (70 °C/5 min), showing the potential of the spray-chilled microcapsules to protect the probiotic from high temperatures and gastrointestinal conditions; in fact, probiotic counts higher than 6 log CFU/g were also shown during storage and after exposure to simulated gastrointestinal conditions compared to the free probiotic.

In conclusion, spray chilling, despite its numerous advantages, still presents challenges that need to be overcome. However, the potential of this technology is considerable, and it proves to be a promising technique for the microencapsulation of probiotics.

### 4.7. Electrospinning and Electrospraying

In recent years, electrohydrodynamic processes, such as electrospinning and electrospraying, have emerged as alternative encapsulation techniques for probiotics [180]. Both electrospinning and electrospraying employ a jet of an electrically charged polymer solution to generate fibres or particles of varying sizes, including micrometre, submicron, and nanoscale. These processes are regarded as “sister technologies” due to their similarities but differ primarily in the concentration of the polymer solution employed and the morphology of the final product [181]. Electrospinning results in the production of fibres, whereas electrospraying yields particles.

**Figure 5:**
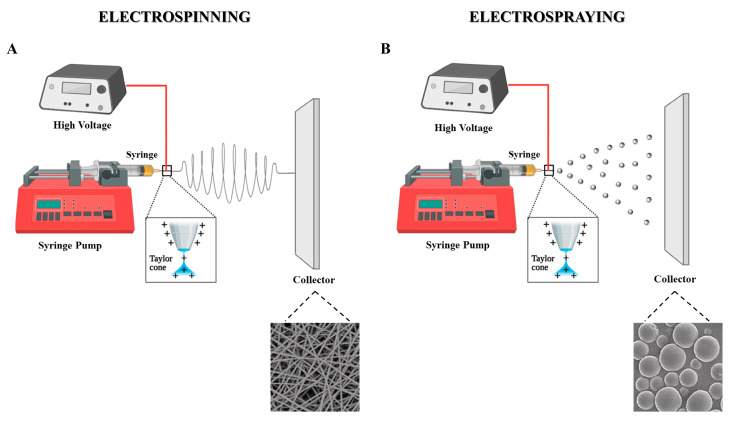
Schematic drawing of setups for electrospinning (**A**) and electrospraying (**B**). Figure licensed under a Creative Commons CC-BY 4.0 license; adapted with permission from [184].

In the process of electrospinning (Figure 5A), a highly concentrated polymer solution is charged by the application of a high electrical voltage, resulting in the generation of free charges. The surface of the droplet at the end of the capillary deforms into a conical shape, known as a Taylor cone, because of the action of electrostatic forces. When the electrostatic force exceeds the surface tension, a charged polymer jet is ejected from the tip of the Taylor cone. The jet then undergoes a whipping motion as it travels towards a grounded manifold, resulting in stretching and the rapid evaporation of the solvent. This process leads to the deposition of thin, solid fibres on the manifold [182].

Electrospraying (Figure 5B), on the other hand, uses a low-concentration polymer solution. The jet from the Taylor cone, formed at the end of the needle due to electrostatic forces, becomes unstable and breaks into fine droplets that disperse. The evaporation of the solvent leads to the contraction and solidification of the droplets, resulting in solid particles deposited on the manifold [183].

These techniques offer several advantages over traditional encapsulation methods. The thin and porous structure of electrospun fibres results in a high surface area per unit mass. This is particularly advantageous for applications such as the immobilisation of enzymes or probiotics, in which an increased surface area results in enhanced efficiency. Both techniques result in no loss of viability during the process, thereby ensuring high encapsulation efficiencies [185]. This is due to the rapid evaporation of the solvent during the process, which traps, for example, probiotics within the fibres or particles. The properties of fibres or particles, including porosity and size, can be modified to facilitate a controlled release of probiotics within the gut, thereby ensuring effective colonisation of the target site [186]. Additionally, both electrospinning and electrospraying can be conducted at room temperature, avoiding the utilisation of high temperatures or aggressive solvents that could potentially damage the probiotics [187].

While electrospinning and electrospraying offer a multitude of benefits, they also present some challenges. Some research [181] has demonstrated a reduction in the viability of probiotic bacteria during the process, which is attributed to the high voltage and shear force applied. Therefore, it is essential to conduct a detailed analysis of the process parameters to minimise these negative effects. One of the primary constraints is the low productivity and costs, which impede the large-scale utilisation of this process. Additionally, the removal phase of the solvent employed may prove challenging, potentially rendering the final product unsuitable for use in the food industry, in which the presence of residual solvents is inadvisable [182]. Furthermore, the recovery and handling of particles present a multitude of challenges. Further research is required to ascertain the viability of incorporating these probiotic formulations into diverse food products, ensuring stability, sensory benefits, and safety.

The techniques of electrospinning and electrospraying have been employed for the microencapsulation of probiotics to enhance their viability and stability within diverse food matrices. This is with a view to the production of fortified foods [188]. Nevertheless, some studies have indicated that the high voltage employed may prove detrimental to cells and potentially impact cell viability. Moayyedi et al. [189] conducted a comparative study of electrospraying, freeze-drying, and atomisation, which revealed that the probiotics obtained via electrospraying were perfectly spherical in shape, in contrast to those produced using other methods. However, regarding viability, electrosprayed probiotics exhibited a greater loss of viability due to cell damage. Conversely, other studies have demonstrated the efficacy of electrospinning and electrospraying for the microencapsulation of different probiotic strains, achieving efficiencies of over 80%. This is due to the careful selection of the wall materials used. Indeed, Gomez-Mascaraque [190] reported a viability loss of *L. plantarum* of less than 1 log CFU/g when using starch mixtures and a whey protein concentrate. Škrlec et al. [191] observed that the incorporation of trehalose and sucrose into PEO resulted in a reduction in damage to *L. plantarum*. In a further study [192], the viability of cells encapsulated in core-shell fibres was found to be greater than that of cells encapsulated in uniaxial fibres due to the addition of FOS. Zaeim et al. [193] conducted a double-layer co-encapsulation of probiotics with inulin using electrospraying, demonstrating enhanced encapsulation yield and elevated survival rates within the GIT. The encapsulation of *Bifidobacterium animalis Bb12* in a whey protein/pulpan concentrate matrix via electrospraying has been demonstrated to be an effective method for creating a protective structure around the probiotics, thereby improving their survival during processing and storage [194]. The use of electrospraying has been demonstrated to encapsulate *Lactobacillus plantarum* in the sodium alginate and pectin matrices, resulting in prolonged viability during refrigeration and providing increased protection to the cells under simulated gastrointestinal conditions and during heat treatment [195]. In additional studies, electrospraying enabled the production of ethylcellulose core-shell microcapsules with high encapsulation efficiencies of the probiotic *Bifidobacterium animalis*, even though the shell matrix had been prepared using solvents that generally substantially reduce their viability [196]. These electrohydrodynamic techniques show promise for producing probiotic-enriched functional foods [197]. Electrospun *L. plantarum* biofilms were shown to possess good reusability and reliability in the production of fermented milk. Furthermore, this milk had a shorter fermentation period and higher survival of probiotics during storage [198]. In another study, kefir was used as a food model to examine the survival of probiotics encapsulated in the electrospun fibre mat [199], demonstrating their improved viability. In another study, electrospun microcapsules encapsulating *L. plantarum* were added to orange juice and ensured high probiotic survival and sensory acceptance of over 88% [200].

Despite the promising potential of electrospraying and electrospinning as microencapsulation technologies for probiotics, these techniques still face significant challenges. Future research should prioritise optimising process parameters to overcome these limitations and fully realise the potential of these technologies for the development of innovative, high-quality, functional foods.

### 4.8. Other Emerging Techniques

#### 4.8.1. Three-Dimensional Printing

Three-dimensional printing represents an emerging technology with promising applications in the food industry [201]. In recent years, researchers have been developing solutions for customised nutrition, adapting to individual dietary needs, allergies, and taste preferences. This approach involves the preparation of personalised food formulas enriched with nutrients and functional compounds to prevent diseases and protect health. Three-dimensional printing makes it possible to use innovative materials, create foods layered with specific nutrients, and improve nutritional quality by replacing unhealthy ingredients with better alternatives. A particular focus is on combining 3D printing with probiotic encapsulation, paving the way for new developments in the field of functional foods with human health benefits. Thanks to these advantages, 3D printing shows great commercial potential in the production of customised foods, enabling the creation of specific products that meet individual needs, with precise control over ingredients and nutritional values.

The increasing demand for functional foods has prompted the development of a food matrix rich in fibre and protein, combined with encapsulation techniques to improve the stability of probiotics during 3D printing [202]. Furthermore, a novel integrated production methodology combining encapsulation, extrusion-based three-dimensional printing, and freeze-drying was devised to develop a storable and cost-effective product that preserves the viability of probiotics, thereby protecting strains such as *Bifidobacterium lactis* and *Lactobacillus acidophilus* [203]. This approach enables the production of snacks or supplements containing live probiotics, with the ability to dose and customise the strain and quantity. Despite progress, some technical challenges remain, such as the stability of probiotics during processing and the selection of suitable materials for food printing. Recent studies have shown that 3D printing can protect probiotics during cooking, with promising results for customised food products. For example, the incorporation of *Lactobacillus plantarum* into wheat products showed high viability, even at high temperatures [204]. An intriguing application is the preparation of functional chocolates with *Lactobacillus plantarum* encapsulated in porous starch. This technique was observed to enhance the survival of probiotics during in vitro digestion. Three-dimensional printing makes it possible to design microcapsules with optimal properties for the protection of probiotics and their incorporation into foods, such as *pasta filata*, that require heat treatment that can exceed 60–70 °C, which is a heat level that could be lethal for beneficial microorganisms if they are not adequately protected. Indeed, these dairy products undergo thermomechanical transformation processes, such as spinning and moulding, which can significantly reduce the viability of the added probiotics. Microencapsulation via 3D printing could be an effective solution to protect the probiotics during these stages and ensure their survival until final consumption.

Three-dimensional printing allows for sensitive bioactive compounds, such as probiotics and antioxidants, to be integrated into customisable food matrices, thereby preserving their functional properties [205]. This approach allows for food to be tailored to specific nutritional needs, reducing waste and optimising time and resources. Due to the possibility of creating complex internal structures, this method represents an opportunity for the development of functional probiotic foods. However, for large-scale industrial applications, it is necessary to balance customisation and standardisation, manage costs, and overcome technical challenges related to ingredient stability and process optimisation. In conclusion, the integration of encapsulation and 3D printing technology has the potential to facilitate the development of innovative and tailor-made functional foods with probiotics selected by strain and dosage. This approach could pave the way for increasingly targeted and customised nutrition, with significant implications for health and well-being.

#### 4.8.2. Microfluidic

**Figure 6:**
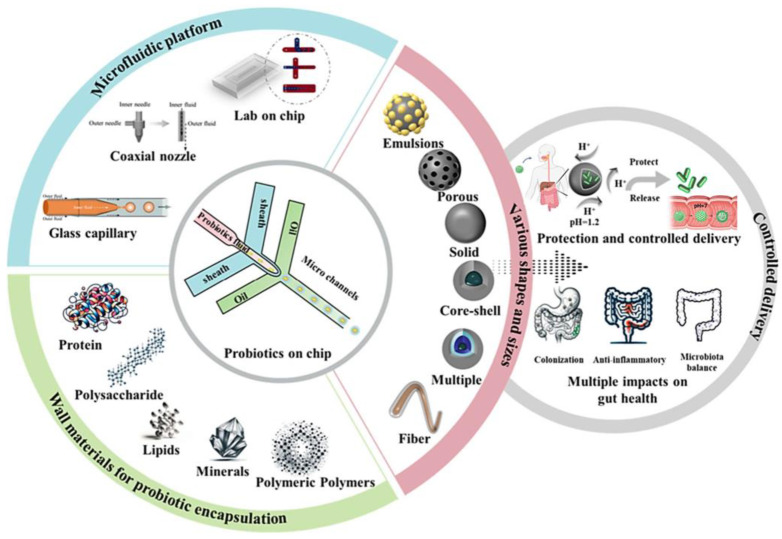
Microfluidic advances in probiotic encapsulation and gastrointestinal delivery. Adapted with permission from [207]. Copyright {2024} American Chemical Society.

Microfluidics (Figure 6) is an innovative technology that involves the precise manipulation of small-volume immiscible fluids within microchannels to achieve precise laminar flow control [206].

The emerging field of microfluidics is proving to be a valuable tool in the microencapsulation of probiotics, offering a diverse range of strategies that can be tailored to the unique requirements of each probiotic, thereby enhancing its stability and efficacy [208]. The microspheres, which are produced with precise control, offer physical protection and targeted intestinal release that is adjustable by modifying the structure of the polymer shell. The use of microfluidics for encapsulating probiotics can overcome some of the limitations of conventional techniques, which can damage the viability of probiotics due to the high temperatures or extreme cold conditions used during the encapsulation process [209]. In contrast, microfluidics offers a gentle method to encapsulate probiotics, preserving their viability, as it avoids the use of high heat or chemical additives [210].

Microfluidics allows for the precise encapsulation of materials and the production of monodispersed particles of uniform size, which is challenging to achieve through traditional encapsulation methods. It also permits accurate control over the shape, size, and internal structure of the encapsulated particles, resulting in high uniformity and reproducibility. Furthermore, microfluidic devices apply reduced shear forces compared to conventional methods during emulsion generation, thereby safeguarding sensitive biomolecules from damage [211]. Microfluidics can achieve higher encapsulation efficiencies compared to traditional methods, minimising the waste of core materials. This technology also enables the production of core-shell microcapsules, opening the door to the creation of particles with complex properties. These particles can not only provide additional protection for probiotics by encapsulating them within a core but also by co-encapsulating them with, for example, prebiotics. This synergistic approach creates a microecosystem that promotes the survival and colonisation of probiotics in the gut. The microfluidic approach ensures greater stability and survival by protecting probiotics from environmental stressors [115]. While large-scale production remains a challenge, microfluidics has the potential to enable automated and controllable large-scale production while maintaining process quality and reducing production costs. Despite its many advantages, microfluidics also has some disadvantages, including the cost of the devices, which limits its applicability in some areas where conventional methods remain less costly.

Numerous studies have shown that microfluidics is a promising technique for the microencapsulation of probiotics. For example, it has enabled the creation of hydrogel microspheres to deliver a probiotic with mucoadhesive properties and colon-targeting capabilities [209]. Another study resulted in the development of double-core microcapsules. The prebiotic shell structure protected the probiotics from gastric fluid, thereby ensuring their release in the intestine. This offers a potential treatment for metabolic syndrome [212]. Furthermore, microfluidics facilitated the production of microspheres containing the probiotic *Bifidobacterium bifidum*, which was prepared from cysteine-modified chitosan. This modification enhanced the adhesion of the probiotics and increased their resistance to gastrointestinal digestion [213]. Finally, studies have explored the use of microfluidics to formulate emulsions that have demonstrated the potential to protect probiotics from environmental and gastrointestinal stresses. This has opened new avenues in the food and nutraceutical industry for the controlled release of probiotics [214].

Overall, microfluidic technology provides a promising platform for the encapsulation and delivery of probiotics. While further research is still required to overcome the current challenges, the future of microfluidics appears promising, with potential for significant advancements and opportunities.

## 5. Characterisation of Probiotic-Loaded Microparticles

After the production of the microparticles using the different techniques analysed above, their characterisation is a crucial step in the probiotic microencapsulation process, as it guarantees the quality and efficacy of the final product.

The first and most important parameter to evaluate is encapsulation efficiency (EE), which measures the effectiveness of trapping and the survival of viable cells during microencapsulation. It represents the percentage of probiotics that have been successfully encapsulated within the microparticles compared to the initial amount present in the feed before the process. A high EE indicates the effective incorporation of probiotics, enhancing their survival during storage and gastrointestinal transit, thereby maximising their health benefits. Careful selection of materials and process optimisation are essential to achieve high EE and preserve the viability and functionality of probiotics. Once the EE value has been determined, the produced microparticles can be characterised through dimensional and morphological analyses. Various microscopy techniques are employed to examine the size, shape, and morphology of the microcapsules, including light microscopy, scanning electron microscopy (SEM), and fluorescence microscopy, the latter of which can differentiate between live and dead bacteria within the capsules [215]. Additionally, dynamic light scattering is a rapid and accurate method for analysing the size distribution of microparticles in suspension [216]. The final size of the microparticles is a critical factor, as it influences their ability to protect and release probiotics. Studies have demonstrated a relationship between the diameter, sphericity, and structural properties (e.g., hardness, cohesion, elasticity, and resilience) of microcapsules and bacterial survival [217]. The physicochemical properties of the microparticles, such as density and porosity, are also important parameters for the processing, storage, packaging, transport, and marketing of the product. Characterisation also involves determining the water activity, moisture content, and hygroscopicity of the microparticles, as these parameters significantly impact the stability of the encapsulated probiotics during storage. Furthermore, it is vital to assess the release profile of the probiotics from the microparticles and their viability in media simulating the harsh conditions of GIT. Another important aspect is the evaluation of thermal stability, which analyses the protective effect of microencapsulation against thermal stress. Finally, assessing the stability of the encapsulated probiotics during storage is essential.

The comprehensive characterisation of microparticles is critical to ensuring the quality and effectiveness of the microencapsulation process. It guarantees that microparticles meet the requirements to protect the probiotics, preserve their viability, and support the development of innovative, high-performance products.

## 6. Conclusions

In conclusion, this review highlights the pivotal role of microencapsulation in addressing the challenges inherent to the incorporation of probiotics into functional dairy products, specifically string cheese. With the growing demand for probiotic-enriched foods, microencapsulation has emerged as a promising solution to safeguard the viability of probiotics during demanding dairy production processes. However, the successful integration of probiotics into dairy products, such as pasta filata, requires overcoming strict temperature constraints, which may jeopardise the stability of probiotic microorganisms. This review provides an in-depth evaluation of the various microencapsulation techniques, each with its own strengths and weaknesses. Techniques such as spray drying, prilling/vibration, and spray congealing are examined for their potential to protect probiotics during dairy processing. Furthermore, this review emphasises the importance of selecting appropriate biocompatible materials for encapsulation, which guarantee not only the stability of the probiotics but also their functionality in the final product. Emerging encapsulation systems are an advanced solution to improve the robustness of probiotics, offering greater protection against harsh dairy environments. However, the challenge of scaling these techniques efficiently for industrial production remains, balancing cost-effectiveness with the need for high-quality results. In summary, although microencapsulation significantly improves the viability, stability, and functionality of probiotics, further research is needed to optimise these techniques and explore new materials that can improve their performance in dairy applications. The transition from laboratory-scale success to industrial-scale production is a major hurdle, the solution to which will depend on advances in production technologies, cost-reduction strategies, and rigorous clinical studies to ensure the safety and efficacy of these probiotics in functional dairy foods. The future of probiotic-enriched dairy products depends on overcoming these challenges and creating opportunities for innovative and effective health foods.

## Figures and Tables

**Table 1:** Commercially available fortified probiotic products [64].

Product/Company	Product Information
Agropur Cooperative (Granby, QC, Canada)	Probiotics encapsulated by sodium alginate bead and incorporated into the dairy products
Ayanda Group As, (Oslo, Norway)	Soft gel capsules contain probiotic bacteria with omega-3 oil (fish oil with DHA/EPA and vitamins)
Bifa-15™ (Eden Foods, Inc., Clinton, MI, USA)	*B. longum* with *Lactobacillus* and oligosaccharide—triple-layer encapsulation—seamless microcapsule delivery system. Contains 3 billion live cells per capsule
Cardioviva™ (Micropharma Inc., Montréal, QC, Canada and Danone Research)	Microencapsulated *L. reuteri* culture in fermented milk
Culturelle^®^ (Cromwell, CT, USA)	Digestive health probiotic capsules contain a minimum of 10 billion live cultures of *Lacticaseibacillus rhamnosus* GG (LGG^®^)
EnCaptimus™ (AnaBio™ Technologies Ltd., Cork, Ireland)	Beverages, gummies, bars, baby foods, sports powder, fruit snacks, and trail mixes
Flying Embers (Fermented Sciences, Inc. and zümXR^®^) (Ventura, CA, USA)	Shelf-stable probiotic hard kombucha—contains a probiotic strain of *Bacillus coagulans* SNZ 1969 and the native kombucha bacteria (Acetobacter)
Mars^®^ Inc. (Hackettstown, NJ, USA)	Low-calorie probiotic milk drink
Micropharma Ltd. (Montréal, QC, Canada)	Sodium alginate beads with multiple surface coatings of poly-LLysine and alginate in some dairy products
PERKii enhanced probiotics (University of Queensland and Sunshine State^®^, Queensland, Australia)	Microencapsulated probiotics using Progel™ technology—bottled with billions of *L. casei* in different fruit flavour drinks
PRO15 Probiotics (Cognoa International Inc., Manila, Philippines)	Probiotic food supplement—contains 11 Lactobacillus and 4 Bifidobacterium Strains, Double microencapsulation technology for protective coating of probiotic strains
Probio’stick^®^ (Montreal, QC, Canada)	Lipid-coated particles (powder form) allow cell release only in the intestine
Probiocap™ Technology (Montréal, QC, Canada)	A typical freeze-dried powder granule is coated with lipids using a fluidised bed spray-coating process
ProbioFerm (Des Moines, IA, USA)	Durabac™ encapsulation technology. Encapsulated powders of individual probiotics with 100 billion CFU/g (*L. acidophilus*, *E. faecium*, *P. acidilactici*, *P. pentosaceus*, *B. bifidum*, *B. longum*, etc.)
ProBiotic bites (Barry Callebaut AG, Zurich, Switzerland)	Chocolate bars containing encapsulated probiotics
UltruBiostix (LosAngeles, CA, USA) and Vitacel^®^Prolac (J. Rettenmaier and Söne, Rosenberg, Germany)	Probiotics encapsulated by soluble and insoluble dietary fibre
YogActive Plus (Yogactive^®^, QC, Canada)	YogActive Probiotic Cereal—probiotics fortified ready-to-eat cereal. Matrix-coated probiotics contain rice, wheat, yoghurt, fruit fibre, and skim milk powder with strawberry/chocolate flavours. Contains 1 billion CFU of *L. acidophilus* LA-5 per serving (33 g)

**Table 2:** Probiotics used in functional dairy foods and their health benefits. Data from [65].

Type of Cheese	Probiotic Microorganism Used	Quantity	Health Benefits	Reference
Cheddar prepared from buffalo milk	*Lactobacillus acidophilus* and *Bifidobacterium bifidum*	8–9 log CFU/g	Compared to the control cheese, the water-soluble extract from probiotic cheddar cheese showed substantially more antithrombotic action.	[66]
Chami	*Pediococcus pentosaceus*	11–12 log CFU/g	During storage, chami enhanced with encapsulated probiotic bacteria showed increased inhibition of α-glucosidase and Dipeptidyl peptidase IV (DPP-IV).	[67]
Prato	*Lacticaseibacillus casei-01*	7–8 log CFU/g	Frequent consumption of probiotic cheese decreased inflammation in the lungs and decreased oxidative stress in the liver, gut, and lungs.	[68]
Cheddar	*Lactobacillus helveticus* 1.0612, *Lacticaseibacillus rhamnosus* 1.0911, *Lacticaseibacillus casei* 1.0319	8–10 log CFU/g	The release of angiotensin-converting (ACE) peptides was facilitated by cheddar cheese containing various microorganisms.	[69]
Kalari	*Lactobacillus plantarum* (NCDC 012), *Lacticaseibacillus casei* (NCDC 297), *Levilactobacillus brevis* (NCDC 021)	6–7 log CFU/g	Kalari cheese is anti-proliferative (against human breast and colon cancer cells, neuroblastoma), antidiabetic, antimicrobial, and immunomodulatory properties were all improved by the addition of probiotics.	[70]
Fresh cheese	*Lactiplantibacillus plantarum* 299v, *Bifidobacterium animalis Bo*	7.5–8.5 log CFU/g	The survival of bacteria in the GIT improved when the strains were paired with the fatty acids in cheese, indicating a possible synergistic effect. Furthermore, the digested fractions enhanced fat breakdown, reduced lipid accumulation in hepatocytes, stimulated adipokine secretion and exhibited anti-inflammatory effects.	[71]
Fresh cheese	*Lactococcus lactis* LB1022, *Lactiplantibacillus plantarum* LB1418	8 log CFU/g	Consuming probiotic cheese reduced liver inflammation, controlled fatty acid oxidation, and enhanced alcohol metabolism.	[72]
Minas Frescal Cheese	*Lactococcus lactis* NCDO 2118	7–8 log CFU/g	Mice that ate the probiotic cheese had less severe colitis, a lower disease activity index, and attenuated weight loss.	[73]
Minas Frescal and Prato (Brazil)	*Lacticaseibacillus casei*-01	8 log CFU/g	It maintained certain glycaemic indices in healthy people and showed stronger antihyperglycemic action in vitro.	[74]

**Table 4:** Examples of dairy functional foods with microencapsulated probiotics produced via spray drying.

Type of Cheese	Probiotic Strain	Encapsulating Material	Main Results	Reference
Cream cheese	*Lactiplantibacillus plantarum* CCMA 0359	Whey powder	High viability at the GIT. It did not alter the organoleptic properties of the cheese.	[161]
Cheddar cheese	*Lb. paracasei* ssp. *paracasei* NFBC 338	Skim milk powder	The probiotic properties are preserved after drying	[154]
Gouda cheese	*Bifidobacterium lactis*	Reconstituted skim milk and a mixture of β-cyclodextrin and gum arabic	The result is a high survival of probiotics in Gouda cheese during ripening and simulated GIconditions	[162]
Iranian white cheese	*Lactiplantibacillus plantarum* ATCC 8014	Whey protein isolate (WPI) and Gum Arabic (GA)	Higher survivability of *L*. *plantarum* ATCC 8014 in freeze-dried microcapsules than in spray-dried microcapsules during storage time (60 days).	[163]
Soft goat cheese	*Lactobacillus plantarum* 564	Skim milk	After 8 weeks of cheese storage, a high level of 8.82 log CFU/g was found for the encapsulated bacteria, while the free-cell number decreased to 6.9 log CFU/g. The addition of spray-dried bacteria did not change the properties of the cheese (pH value, chemical composition, sensory quality).	[164]

## References

[1] Guarner F., Perdigon G., Corthier G., Salminen S., Koletzko B., Morelli L. (2005). Should Yoghurt Cultures Be Considered Probiotic?. Br. J. Nutr..

[2] Lilly D.M., Stillwell R.H. (1965). Probiotics: Growth-Promoting Factors Produced by Microorganisms. Science.

[3] Fuller R. (1989). Probiotics in Man and Animals. J. Appl. Bacteriol..

[4] Hill C., Guarner F., Reid G., Gibson G.R., Merenstein D.J., Pot B., Morelli L., Canani R.B., Flint H.J., Salminen S. (2014). The International Scientific Association for Probiotics and Prebiotics Consensus Statement on the Scope and Appropriate Use of the Term Probiotic. Nat. Rev. Gastroenterol. Hepatol..

[5] Zheng J., Wittouck S., Salvetti E., Franz C.M.A.P., Harris H.M.B., Mattarelli P., O’Toole P.W., Pot B., Vandamme P., Walter J. (2020). A Taxonomic Note on the Genus Lactobacillus: Description of 23 Novel Genera, Emended Description of the Genus Lactobacillus Beijerinck 1901, and Union of Lactobacillaceae and Leuconostocaceae. Int. J. Syst. Evol. Microbiol..

[6] Fijan S. (2014). Microorganisms with Claimed Probiotic Properties: An Overview of Recent Literature. Int. J. Environ. Res. Public Health.

[7] Ministero Della Salute Direzione Generale per l’igiene e La Sicurezza Degli Alimenti e La Nutrizione- Ufficio 4 GUIDELINES ON PROBIOTICS AND PREBIOTICS Revised in March 2018. https://www.salute.gov.it/imgs/C_17_pubblicazioni_1016_ulterioriallegati_ulterioreallegato_0_alleg.pdf.

[8] Celano G., Calabrese F.M., Riezzo G., D’Attoma B., Ignazzi A., Di Chito M., Sila A., De Nucci S., Rinaldi R., Linsalata M. (2024). A Multi-Omics Approach to Disclose Metabolic Pathways Impacting Intestinal Permeability in Obese Patients Undergoing Very Low Calorie Ketogenic Diet. Nutrients.

[9] Quigley E.M.M. (2019). Prebiotics and Probiotics in Digestive Health. Clin. Gastroenterol. Hepatol..

[10] Mazziotta C., Tognon M., Martini F., Torreggiani E., Rotondo J.C. (2023). Probiotics Mechanism of Action on Immune Cells and Beneficial Effects on Human Health. Cells.

[11] Rondanelli M., Faliva M.A., Perna S., Giacosa A., Peroni G., Castellazzi A.M. (2017). Using Probiotics in Clinical Practice: Where Are We Now? A Review of Existing Meta-Analyses. Gut Microbes.

[12] McFarland L.V., Karakan T., Karatas A. (2021). Strain-Specific and Outcome-Specific Efficacy of Probiotics for the Treatment of Irritable Bowel Syndrome: A Systematic Review and Meta-Analysis. EClinicalMedicine.

[13] McFarland L.V., Evans C.T., Goldstein E.J.C. (2018). Strain-Specificity and Disease-Specificity of Probiotic Efficacy: A Systematic Review and Meta-Analysis. Front. Med..

[14] Merenstein D.J., Tancredi D.J., Karl J.P., Krist A.H., Lenoir-Wijnkoop I., Reid G., Roos S., Szajewska H., Sanders M.E. (2024). Is There Evidence to Support Probiotic Use for Healthy People?. Adv. Nutr..

[15] Parker E.A., Roy T., D’Adamo C.R., Wieland L.S. (2018). Probiotics and Gastrointestinal Conditions: An Overview of Evidence from the Cochrane Collaboration. Nutrition.

[16] Kowalczyk M., Radziwill-Bienkowska J.M., Marć M.A., Jastrząb R., Mytych J., Siedlecki P., Szczepankowska A.K. (2024). Screening for Probiotic Properties and Potential Immunogenic Effects of Lactobacilli Strains Isolated from Various Food Products. Front. Microbiol..

[17] Gu S., Yang D., Liu C., Xue W. (2023). The Role of Probiotics in Prevention and Treatment of Food Allergy. Food Sci. Human Wellness.

[18] Aghamohammad S., Sepehr A., Miri S.T., Najafi S., Pourshafie M.R., Rohani M. (2023). Investigation of the Anti-Inflammatory Effects of Native Potential Probiotics as Supplementary Therapeutic Agents in an in-Vitro Model of Inflammation. BMC Complement. Med. Ther..

[19] Wolvers D., Antoine J.-M., Myllyluoma E., Schrezenmeir J., Szajewska H., Rijkers G.T. (2010). Guidance for Substantiating the Evidence for Beneficial Effects of Probiotics: Prevention and Management of Infections by Probiotics. J. Nutr..

[20] Reid G., Younes J.A., Van der Mei H.C., Gloor G.B., Knight R., Busscher H.J. (2011). Microbiota Restoration: Natural and Supplemented Recovery of Human Microbial Communities. Nat. Rev. Microbiol..

[21] Kumar M., Nagpal R., Verma V., Kumar A., Kaur N., Hemalatha R., Gautam S.K., Singh B. (2013). Probiotic Metabolites as Epigenetic Targets in the Prevention of Colon Cancer. Nutr. Rev..

[22] van Baarlen P., Troost F., van der Meer C., Hooiveld G., Boekschoten M., Brummer R.J.M., Kleerebezem M. (2011). Human Mucosal in Vivo Transcriptome Responses to Three Lactobacilli Indicate How Probiotics May Modulate Human Cellular Pathways. Proc. Natl. Acad. Sci. USA.

[23] De Angelis M., Siragusa S., Vacca M., Di Cagno R., Cristofori F., Schwarm M., Pelzer S., Flügel M., Speckmann B., Francavilla R. (2021). Selection of Gut-Resistant Bacteria and Construction of Microbial Consortia for Improving Gluten Digestion under Simulated Gastrointestinal Conditions. Nutrients.

[24] Ritchie M.L., Romanuk T.N. (2012). A Meta-Analysis of Probiotic Efficacy for Gastrointestinal Diseases. PLoS ONE.

[25] EFSA Panel on Dietetic Products, Nutrition and Allergies (NDA) (2010). Scientific Opinion on the Substantiation of Health Claims Related to Live Yoghurt Cultures and Improved Lactose Digestion (ID 1143, 2976) Pursuant to Article 13(1) of Regulation (EC) No 1924/2006. EFSA J..

[26] Gibson G.R., Probert H.M., Van Loo J., Rastall R.A., Roberfroid M.B. (2004). Dietary Modulation of the Human Colonic Microbiota: Updating the Concept of Prebiotics. Nutr. Res. Rev..

[27] Gibson G.R., Hutkins R., Sanders M.E., Prescott S.L., Reimer R.A., Salminen S.J., Scott K., Stanton C., Swanson K.S., Cani P.D. (2017). Expert Consensus Document: The International Scientific Association for Probiotics and Prebiotics (ISAPP) Consensus Statement on the Definition and Scope of Prebiotics. Nat. Rev. Gastroenterol. Hepatol..

[28] Swanson K.S., Gibson G.R., Hutkins R., Reimer R.A., Reid G., Verbeke K., Scott K.P., Holscher H.D., Azad M.B., Delzenne N.M. (2020). The International Scientific Association for Probiotics and Prebiotics (ISAPP) Consensus Statement on the Definition and Scope of Synbiotics. Nat. Rev. Gastroenterol. Hepatol..

[29] Lyon J., Connell M., Chandrasekaran K., Srivastava S. (2023). Effect of Synbiotics on Weight Loss and Metabolic Health in Adults with Overweight and Obesity: A Randomized Controlled Trial. Obesity.

[30] Álvarez-Arraño V., Martín-Peláez S. (2021). Effects of Probiotics and Synbiotics on Weight Loss in Subjects with Overweight or Obesity: A Systematic Review. Nutrients.

[31] Naseri K., Saadati S., Ashtary-Larky D., Asbaghi O., Ghaemi F., Pashayee-Khamene F., Yari Z., de Courten B. (2022). Probiotics and Synbiotics Supplementation Improve Glycemic Control Parameters in Subjects with Prediabetes and Type 2 Diabetes Mellitus: A GRADE-Assessed Systematic Review, Meta-Analysis, and Meta-Regression of Randomized Clinical Trials. Pharmacol. Res..

[32] Rong L., Ch’ng D., Jia P., Tsoi K.K.F., Wong S.H., Sung J.J.Y. (2023). Use of Probiotics, Prebiotics, and Synbiotics in Non-alcoholic Fatty Liver Disease: A Systematic Review and Meta-analysis. J. Gastroenterol. Hepatol..

[33] Zhang W.X., Shi L.B., Zhou M.S., Wu J., Shi H.Y. (2023). Efficacy of Probiotics, Prebiotics and Synbiotics in Irritable Bowel Syndrome: A Systematic Review and Meta-Analysis of Randomized, Double-Blind, Placebo-Controlled Trials. J. Med. Microbiol..

[34] Veziant J., Bonnet M., Occean B.V., Dziri C., Pereira B., Slim K. (2022). Probiotics/Synbiotics to Reduce Infectious Complications after Colorectal Surgery: A Systematic Review and Meta-Analysis of Randomised Controlled Trials. Nutrients.

[35] Vacca M., Celano G., Calabrese F.M., Rocchetti M.T., Iacobellis I., Serale N., Calasso M., Gesualdo L., De Angelis M. (2023). In Vivo Evaluation of an Innovative Synbiotics on Stage IIIb-IV Chronic Kidney Disease Patients. Front. Nutr..

[36] Choy C.T., Siu P.L.K., Zhou J., Wong C.H., Lee Y.W., Chan H.W., Tsui J.C.C., Lo C.J.Y., Loo S.K.F., Tsui S.K.W. (2023). Improvements in Gut Microbiome Composition Predict the Clinical Efficacy of a Novel Synbiotics Formula in Children with Mild to Moderate Atopic Dermatitis. Microorganisms.

[37] Wendel U. (2022). Assessing Viability and Stress Tolerance of Probiotics—A Review. Front. Microbiol..

[38] Bezkorovainy A. (2001). Probiotics: Determinants of Survival and Growth in the Gut. Am. J. Clin. Nutr..

[39] Champagne C.P., Gardner N.J., Roy D. (2005). Challenges in the Addition of Probiotic Cultures to Foods. Crit. Rev. Food Sci. Nutr..

[40] Guimarães J.T., Balthazar C.F., Silva R., Rocha R.S., Graça J.S., Esmerino E.A., Silva M.C., Sant’Ana A.S., Duarte M.C.K.H., Freitas M.Q. (2020). Impact of Probiotics and Prebiotics on Food Texture. Curr. Opin. Food Sci..

[41] Calasso M., Marzano M., Caponio G.R., Celano G., Fosso B., Calabrese F.M., De Palma D., Vacca M., Notario E., Pesole G. (2023). Shelf-Life Extension of Leavened Bakery Products by Using Bio-Protective Cultures and Type-III Sourdough. LWT.

[42] do Espírito Santo A.P., Perego P., Converti A., Oliveira M.N. (2011). Influence of Food Matrices on Probiotic Viability—A Review Focusing on the Fruity Bases. Trends Food Sci. Technol..

[43] Kathiriya M.R., Vekariya Y.V., Hati S. (2023). Understanding the Probiotic Bacterial Responses Against Various Stresses in Food Matrix and Gastrointestinal Tract: A Review. Probiotics Antimicrob. Proteins.

[44] Fiocco D., Longo A., Arena M.P., Russo P., Spano G., Capozzi V. (2020). How Probiotics Face Food Stress: They Get by with a Little Help. Crit. Rev. Food Sci. Nutr..

[45] Gagnaire V., Jardin J., Rabah H., Briard-Bion V., Jan G. (2015). Emmental Cheese Environment Enhances Propionibacterium Freudenreichii Stress Tolerance. PLoS ONE.

[46] Mangiagalli M., Sarusi G., Kaleda A., Bar Dolev M., Nardone V., Vena V.F., Braslavsky I., Lotti M., Nardini M. (2018). Structure of a Bacterial Ice Binding Protein with Two Faces of Interaction with Ice. FEBS J..

[47] Song S., Bae D.-W., Lim K., Griffiths M.W., Oh S. (2014). Cold Stress Improves the Ability of Lactobacillus Plantarum L67 to Survive Freezing. Int. J. Food Microbiol..

[48] Polo L., Mañes-Lázaro R., Olmeda I., Cruz-Pio L.E., Medina Á., Ferrer S., Pardo I. (2017). Influence of Freezing Temperatures Prior to Freeze-Drying on Viability of Yeasts and Lactic Acid Bacteria Isolated from Wine. J. Appl. Microbiol..

[49] Ferrando V., Quiberoni A., Reinheimer J., Suárez V. (2016). Functional Properties of Lactobacillus Plantarum Strains: A Study in Vitro of Heat Stress Influence. Food Microbiol..

[50] Papadimitriou K., Alegría Á., Bron P.A., de Angelis M., Gobbetti M., Kleerebezem M., Lemos J.A., Linares D.M., Ross P., Stanton C. (2016). Stress Physiology of Lactic Acid Bacteria. Microbiol. Mol. Biol. Rev..

[51] Dixon S.J., Stockwell B.R. (2014). The Role of Iron and Reactive Oxygen Species in Cell Death. Nat. Chem. Biol..

[52] Feng T., Wang J. (2020). Oxidative Stress Tolerance and Antioxidant Capacity of Lactic Acid Bacteria as Probiotic: A Systematic Review. Gut Microbes.

[53] Bisson G., Maifreni M., Innocente N., Marino M. (2023). Application of Pre-Adaptation Strategies to Improve the Growth of Probiotic Lactobacilli under Food-Relevant Stressful Conditions. Food Funct..

[54] Castro-López C., Romero-Luna H.E., García H.S., Vallejo-Cordoba B., González-Córdova A.F., Hernández-Mendoza A. (2023). Key Stress Response Mechanisms of Probiotics During Their Journey Through the Digestive System: A Review. Probiotics Antimicrob. Proteins.

[55] Onwe R.O., Onwosi C.O., Ezugworie F.N., Ekwealor C.C., Okonkwo C.C. (2022). Microbial Trehalose Boosts the Ecological Fitness of Biocontrol Agents, the Viability of Probiotics during Long-Term Storage and Plants Tolerance to Environmental-Driven Abiotic Stress. Sci. Total Environ..

[56] Tripathi M.K., Giri S.K. (2014). Probiotic Functional Foods: Survival of Probiotics during Processing and Storage. J. Funct. Foods.

[57] Soares M.B., Martinez R.C.R., Pereira E.P.R., Balthazar C.F., Cruz A.G., Ranadheera C.S., Sant’Ana A.S. (2019). The Resistance of Bacillus, Bifidobacterium, and Lactobacillus Strains with Claimed Probiotic Properties in Different Food Matrices Exposed to Simulated Gastrointestinal Tract Conditions. Food Res. Int..

[58] Naissinger da Silva M., Tagliapietra B.L., Flores V.d.A., Pereira dos Santos Richards N.S. (2021). In Vitro Test to Evaluate Survival in the Gastrointestinal Tract of Commercial Probiotics. Curr. Res. Food Sci..

[59] Ruiz L., Margolles A., Sánchez B. (2013). Bile Resistance Mechanisms in Lactobacillus and Bifidobacterium. Front. Microbiol..

[60] FAO (2006). Probiotics in Food Health and Nutritional Properties and Guidelines for Evaluation.

[61] Kaur H., Kaur G., Ali S.A. (2022). Dairy-Based Probiotic-Fermented Functional Foods: An Update on Their Health-Promoting Properties. Fermentation.

[62] Temple N.J. (2022). A Rational Definition for Functional Foods: A Perspective. Front. Nutr..

[63] De Prisco A., Mauriello G. (2016). Probiotication of Foods: A Focus on Microencapsulation Tool. Trends Food Sci. Technol..

[64] Yoha K.S., Nida S., Dutta S., Moses J.A., Anandharamakrishnan C. (2022). Targeted Delivery of Probiotics: Perspectives on Research and Commercialization. Probiotics Antimicrob. Proteins.

[65] Araujo H.C.S., de Jesus M.S., Sandes R.D.D., Leite Neta M.T.S., Narain N. (2024). Functional Cheeses: Updates on Probiotic Preservation Methods. Fermentation.

[66] Anees Ur Rehman M., Ashfaq K., Ashfaq T., Abuzar Ghaffari M., Ali N., Kazmi F., Sohail N. (2022). The Antithrombotic Potential of Bioactive Peptides Induced by Buffalo Milk Probiotic Cheddar Cheese. Pak. BioMed. J..

[67] Mudgil P., Aldhaheri F., Hamdi M., Punia S., Maqsood S. (2022). Fortification of Chami (Traditional Soft Cheese) with Probiotic-Loaded Protein and Starch Microparticles: Characterization, Bioactive Properties, and Storage Stability. LWT.

[68] Vasconcelos F.M., Silva H.L.A., Poso S.M.V., Barroso M.V., Lanzetti M., Rocha R.S., Graça J.S., Esmerino E.A., Freitas M.Q., Silva M.C. (2019). Probiotic Prato Cheese Attenuates Cigarette Smoke-Induced Injuries in Mice. Food Res. Int..

[69] Hao X., Yang W., Zhu Q., Zhang G., Zhang X., Liu L., Li X., Hussain M., Ni C., Jiang X. (2021). Proteolysis and ACE-Inhibitory Peptide Profile of Cheddar Cheese: Effect of Digestion Treatment and Different Probiotics. LWT.

[70] Mushtaq M., Gani A., Masoodi F.A. (2019). Himalayan Cheese (Kalari/Kradi) Fermented with Different Probiotic Strains: In Vitro Investigation of Nutraceutical Properties. LWT.

[71] Machado M., Sousa S.C., Rodríguez-Alcalá L.M., Pintado M., Gomes A.M. (2023). Functional Lipid Enriched Probiotic Cheese: Gastrointestinal Stability and Potential Health Benefits. Int. Dairy J..

[72] Kim J.-H., Woo D., Nam Y., Baek J., Lee J.-Y., Kim W. (2023). Probiotic Cheese Improves Alcohol Metabolism and Alleviates Alcohol-Induced Liver Injury via the SIRT1/AMPK Signaling Pathway. J. Funct. Foods.

[73] Cordeiro B.F., Alves J.L., Belo G.A., Oliveira E.R., Braga M.P., da Silva S.H., Lemos L., Guimarães J.T., Silva R., Rocha R.S. (2021). Therapeutic Effects of Probiotic Minas Frescal Cheese on the Attenuation of Ulcerative Colitis in a Murine Model. Front. Microbiol..

[74] Grom L.C., Rocha R.S., Balthazar C.F., Guimarães J.T., Coutinho N.M., Barros C.P., Pimentel T.C., Venâncio E.L., Collopy Junior I., Maciel P.M.C. (2020). Postprandial Glycemia in Healthy Subjects: Which Probiotic Dairy Food Is More Adequate?. J. Dairy Sci..

[75] Adhikari K., Mustapha A., Grün I.U., Fernando L. (2000). Viability of Microencapsulated Bifidobacteria in Set Yogurt During Refrigerated Storage. J. Dairy Sci..

[76] Wang M., Wang C., Gao F., Guo M. (2018). Effects of Polymerised Whey Protein-Based Microencapsulation on Survivability of Lactobacillus Acidophilus LA-5 and Physiochemical Properties of Yoghurt. J. Microencapsul..

[77] Patrignani F., Siroli L., Serrazanetti D.I., Braschi G., Betoret E., Reinheimer J.A., Lanciotti R. (2017). Microencapsulation of Functional Strains by High Pressure Homogenization for a Potential Use in Fermented Milk. Food Res. Int..

[78] Kavas N., Kavas G., Kinik Ö., Ateş M., Kaplan M., Şatir G. (2022). Symbiotic Microencapsulation to Enhance Bifidobacterium Longum and Lactobacillus Paracasei Survival in Goat Cheese. Food Sci. Technol..

[79] Reale A., Di Renzo T., Coppola R. (2019). Factors Affecting Viability of Selected Probiotics during Cheese-Making of Pasta Filata Dairy Products Obtained by Direct-to-Vat Inoculation System. LWT.

[80] Angiolillo L., Conte A., Faccia M., Zambrini A.V., Del Nobile M.A. (2014). A New Method to Produce Synbiotic Fiordilatte Cheese. Innov. Food Sci. Emerg. Technol..

[81] Vacca M., Celano G., Serale N., Costantino G., Calabrese F.M., Calasso M., De Angelis M. (2024). Dynamic Microbial and Metabolic Changes during Apulian Caciocavallo Cheesemaking and Ripening Produced According to a Standardized Protocol. J. Dairy Sci..

[82] Bihola A., Sharma H., Chaudhary M.B., Bumbadiya M.R., Kumar D., Adil S. (2024). Recent Developments in Cheese Technologies. Food Rev. Int..

[83] Minervini F., Siragusa S., Faccia M., Dal Bello F., Gobbetti M., De Angelis M. (2012). Manufacture of Fior Di Latte Cheese by Incorporation of Probiotic Lactobacilli. J. Dairy Sci..

[84] Cuffia F., George G., Godoy L., Vinderola G., Reinheimer J., Burns P. (2019). In Vivo Study of the Immunomodulatory Capacity and the Impact of Probiotic Strains on Physicochemical and Sensory Characteristics: Case of Pasta Filata Soft Cheeses. Food Res. Int..

[85] Akarca G., Yildirim G. (2022). Effects of the Probiotic Bacteria on the Quality Properties of Mozzarella Cheese Produced from Different Milk. J. Food Sci. Technol..

[86] Albenzio M., Santillo A., Caroprese M., Braghieri A., Sevi A., Napolitano F. (2013). Composition and Sensory Profiling of Probiotic Scamorza Ewe Milk Cheese. J. Dairy Sci..

[87] Alsaleem K., Hamouda M., Alayouni R., Elfaruk M., Hammam A. (2023). Effect of Skim Milk Powder and Whey Protein Concentrate Addition on the Manufacture of Probiotic Mozzarella Cheese. Fermentation.

[88] Ortakci F., Broadbent J.R., McManus W.R., McMahon D.J. (2012). Survival of Microencapsulated Probiotic Lactobacillus Paracasei LBC-1e during Manufacture of Mozzarella Cheese and Simulated Gastric Digestion. J. Dairy Sci..

[89] Mukhtar H., Yaqub S., Haq I. (2020). ul Production of Probiotic Mozzarella Cheese by Incorporating Locally Isolated Lactobacillus Acidophilus. Ann. Microbiol..

[90] Minervini F., Conte A., Del Nobile M.A., Gobbetti M., De Angelis M. (2017). Dietary Fibers and Protective Lactobacilli Drive Burrata Cheese Microbiome. Appl. Environ. Microbiol..

[91] Cook M.T., Tzortzis G., Charalampopoulos D., Khutoryanskiy V.V. (2012). Microencapsulation of Probiotics for Gastrointestinal Delivery. J. Control. Release.

[92] Dodoo C.C., Wang J., Basit A.W., Stapleton P., Gaisford S. (2017). Targeted Delivery of Probiotics to Enhance Gastrointestinal Stability and Intestinal Colonisation. Int. J. Pharm..

[93] Sbehat M., Mauriello G., Altamimi M. (2022). Microencapsulation of Probiotics for Food Functionalization: An Update on Literature Reviews. Microorganisms.

[94] McClements D.J. (2014). Nanoparticle- and Microparticle-Based Delivery Systems.

[95] Misra S., Pandey P., Dalbhagat C.G., Mishra H.N. (2022). Emerging Technologies and Coating Materials for Improved Probiotication in Food Products: A Review. Food Bioprocess Technol..

[96] Vivek K., Mishra S., Pradhan R.C., Nagarajan M., Kumar P.K., Singh S.S., Manvi D., Gowda N.N. (2023). A Comprehensive Review on Microencapsulation of Probiotics: Technology, Carriers and Current Trends. Appl. Food Res..

[97] Arepally D., Reddy R.S., Goswami T.K., Coorey R. (2022). A Review on Probiotic Microencapsulation and Recent Advances of Their Application in Bakery Products. Food Bioprocess Technol..

[98] Razavi S., Janfaza S., Tasnim N., Gibson D.L., Hoorfar M. (2021). Microencapsulating Polymers for Probiotics Delivery Systems: Preparation, Characterization, and Applications. Food Hydrocoll..

[99] Kowalska E., Ziarno M., Ekielski A., Żelaziński T. (2022). Materials Used for the Microencapsulation of Probiotic Bacteria in the Food Industry. Molecules.

[100] Terpou A., Papadaki A., Lappa I., Kachrimanidou V., Bosnea L., Kopsahelis N. (2019). Probiotics in Food Systems: Significance and Emerging Strategies Towards Improved Viability and Delivery of Enhanced Beneficial Value. Nutrients.

[101] Mahmoud M., Abdallah N.A., El-Shafei K., Tawfik N.F., El-Sayed H.S. (2020). Survivability of Alginate-Microencapsulated Lactobacillus Plantarum during Storage, Simulated Food Processing and Gastrointestinal Conditions. Heliyon.

[102] Whelehan M., Marison I.W. (2011). Microencapsulation Using Vibrating Technology. J. Microencapsul..

[103] Ivone M., Denora N., D’Amico V., Mareczek L., Mueller L.K., Arduino I., Ambruosi A., Lopedota A.A. (2024). Microbeads Produced by Prilling/Vibration Technique: A New Way to Use Polyvinyl Alcohol in Pediatric and Veterinary Formulations. J. Drug Deliv. Sci. Technol..

[104] D’Amico V., Denora N., Ivone M., Iacobazzi R.M., Laquintana V., Cutrignelli A., Franco M., Barone M., Lopalco A., Lopedota A.A. (2024). Investigating the Prilling/Vibration Technique to Produce Gastric-Directed Drug Delivery Systems for Misoprostol. Int. J. Pharm..

[105] Bennacef C., Desobry S., Probst L., Desobry-Banon S. (2023). Alginate Based Core–Shell Capsules Production through Coextrusion Methods: Recent Applications. Foods.

[106] Lopalco A., Denora N., Laquintana V., Cutrignelli A., Franco M., Robota M., Hauschildt N., Mondelli F., Arduino I., Lopedota A. (2020). Taste Masking of Propranolol Hydrochloride by Microbeads of EUDRAGIT® E PO Obtained with Prilling Technique for Paediatric Oral Administration. Int. J. Pharm..

[107] Lopedota A.A., Arduino I., Lopalco A., Iacobazzi R.M., Cutrignelli A., Laquintana V., Racaniello G.F., Franco M., la Forgia F., Fontana S. (2021). From Oil to Microparticulate by Prilling Technique: Production of Polynucleate Alginate Beads Loading Serenoa Repens Oil as Intestinal Delivery Systems. Int. J. Pharm..

[108] Santillo A., Albenzio M., Bevilacqua A., Corbo M.R., Sevi A. (2012). Encapsulation of Probiotic Bacteria in Lamb Rennet Paste: Effects on the Quality of Pecorino Cheese. J. Dairy Sci..

[109] Eckert C., Agnol W.D., Dallé D., Serpa V.G., Maciel M.J., Lehn D.N., Volken de Souza C.F. (2018). Development of Alginate-Pectin Microparticles with Dairy Whey Using Vibration Technology: Effects of Matrix Composition on the Protection of Lactobacillus Spp. from Adverse Conditions. Food Res. Int..

[110] D’Amico V., Lopalco A., Iacobazzi R.M., Vacca M., Siragusa S., De Angelis M., Lopedota A.A., Denora N. (2024). Multistimuli Responsive Microcapsules Produced by the Prilling/Vibration Technique for Targeted Colonic Delivery of Probiotics. Int. J. Pharm..

[111] Gu Q., Yin Y., Yan X., Liu X., Liu F., McClements D.J. (2022). Encapsulation of Multiple Probiotics, Synbiotics, or Nutrabiotics for Improved Health Effects: A Review. Adv. Colloid Interface Sci..

[112] Krasaekoopt W., Bhandari B., Deeth H. (2003). Evaluation of Encapsulation Techniques of Probiotics for Yoghurt. Int. Dairy J..

[113] Silva C.M., Ribeiro A.J., Figueiredo I.V., Gonçalves A.R., Veiga F. (2006). Alginate Microspheres Prepared by Internal Gelation: Development and Effect on Insulin Stability. Int. J. Pharm..

[114] Burgain J., Gaiani C., Linder M., Scher J. (2011). Encapsulation of Probiotic Living Cells: From Laboratory Scale to Industrial Applications. J. Food Eng..

[115] Camelo-Silva C., Verruck S., Ambrosi A., Di Luccio M. (2022). Innovation and Trends in Probiotic Microencapsulation by Emulsification Techniques. Food Eng. Rev..

[116] Das A., Ray S., Raychaudhuri U., Chakraborty R. (2014). Microencapsulation of Probiotic Bacteria and Its Potential Application in Food Technology. Int. J. Agric. Environ. Biotechnol..

[117] El Sayed H.S., Mabrouk A.M. (2023). Encapsulation of Probiotics Using Mixed Sodium Alginate and Rice Flour to Enhance Their Survivability in Simulated Gastric Conditions and in UF-Kariesh Cheese. Biocatal. Agric. Biotechnol..

[118] Al-Moghazy M., El-Sayed H.S., Abo-Elwafa G.A. (2022). Co-Encapsulation of Probiotic Bacteria, Fish Oil and Pomegranate Peel Extract for Enhanced White Soft Cheese. Food Biosci..

[119] Lopes L.A.A., Pimentel T.C., Carvalho R.d.S.F., Madruga M.S., de Sousa Galvão M., Bezerra T.K.A., Barão C.E., Magnani M., Stamford T.C.M. (2021). Spreadable Goat Ricotta Cheese Added with Lactobacillus Acidophilus La-05: Can Microencapsulation Improve the Probiotic Survival and the Quality Parameters?. Food Chem..

[120] Rodríguez-Huezo M.E., Estrada-Fernández A.G., García-Almendárez B.E., Ludeña-Urquizo F., Campos-Montiel R.G., Pimentel-González D.J. (2014). Viability of Lactobacillus Plantarum Entrapped in Double Emulsion during Oaxaca Cheese Manufacture, Melting and Simulated Intestinal Conditions. LWT-Food Sci. Technol..

[121] Koh W.Y., Lim X.X., Tan T.-C., Kobun R., Rasti B. (2022). Encapsulated Probiotics: Potential Techniques and Coating Materials for Non-Dairy Food Applications. Appl. Sci..

[122] Zhang R., Hoffmann T., Tsotsas E. (2020). Novel Technique for Coating of Fine Particles Using Fluidized Bed and Aerosol Atomizer. Processes.

[123] Sánchez-Portilla Z., Melgoza-Contreras L.M., Reynoso-Camacho R., Pérez-Carreón J.I., Gutiérrez-Nava A. (2020). Incorporation of Bifidobacterium Sp. into Powder Products through a Fluidized Bed Process for Enteric Targeted Release. J. Dairy Sci..

[124] Mirzamani S.S., Bassiri A.R., Tavakolipour H., Azizi M.H., Kargozari M. (2021). Survival of Fluidized Bed Encapsulated Lactobacillus Acidophilus under Simulated Gastro-Intestinal Conditions and Heat Treatment during Bread Baking. J. Food Meas. Charact..

[125] Azim H., Kalavathy R., Julianto T., Sieo C.C., Ho Y.W. (2012). Effect of Heat, PH and Coating Process with Stearic Acid Using a Fluidized Bed Granulator on Viability of Probiotic Lactobacillus Reuteri C 10. Afr. J. Biotechnol..

[126] Mohylyuk V., Patel K., Scott N., Richardson C., Murnane D., Liu F. (2020). Wurster Fluidised Bed Coating of Microparticles: Towards Scalable Production of Oral Sustained-Release Liquid Medicines for Patients with Swallowing Difficulties. AAPS PharmSciTech.

[127] Hathi Z., Mettu S., Priya A., Athukoralalage S., Lam T.N., Choudhury N.R., Dutta N.K., El-Omar E.M., Gong L., Mohan G. (2021). Methodological Advances and Challenges in Probiotic Bacteria Production: Ongoing Strategies and Future Perspectives. Biochem. Eng. J..

[128] Galvão A.M.M.T., Rodrigues S., Fernandes F.A.N. (2020). Probiotic Dried Apple Snacks: Development of Probiotic Coating and Shelf-life Studies. J. Food Process Preserv..

[129] Mirzamani S.S., Bassiri A., Tavakolipour H., Azizi M.H., Kargozari M. (2021). Fluidized Bed Microencapsulation of Lactobacillus Sporogenes with Some Selected Hydrocolloids for Probiotic Bread Production. J. Food Biosci. Technol..

[130] Lapsiri W., Bhandari B., Wanchaitanawong P. (2012). Viability of *Lactobacillus Plantarum* TISTR 2075 in Different Protectants during Spray Drying and Storage. Dry. Technol..

[131] Kumar S.K., Jayaprakasha H.M., Paik H.-D., Kim S.-K., Han S.-E., Jeong A.-R., Yoon Y.-C. (2010). Production of Ready-to-Reconstitute Functional Beverages by Utilizing Whey Protein Hydrolysates and Probiotics. Korean J. Food Sci. Anim. Resour..

[132] Jiang J., Ma C., Song X., Zeng J., Zhang L., Gong P. (2022). Spray Drying Co-Encapsulation of Lactic Acid Bacteria and Lipids: A Review. Trends Food Sci. Technol..

[133] Assegehegn G., Brito-de la Fuente E., Franco J.M., Gallegos C. (2019). The Importance of Understanding the Freezing Step and Its Impact on Freeze-Drying Process Performance. J. Pharm. Sci..

[134] Rokka S., Rantamäki P. (2010). Protecting Probiotic Bacteria by Microencapsulation: Challenges for Industrial Applications. Eur. Food Res. Technol..

[135] Coghetto C.C., Brinques G.B., Ayub M.A.Z. (2016). Probiotics Production and Alternative Encapsulation Methodologies to Improve Their Viabilities under Adverse Environmental Conditions. Int. J. Food Sci. Nutr..

[136] Azam M., Saeed M., Pasha I., Shahid M. (2020). A Prebiotic-Based Biopolymeric Encapsulation System for Improved Survival of Lactobacillus Rhamnosus. Food Biosci..

[137] O’Riordan K., Andrews D., Buckle K., Conway P. (2001). Evaluation of Microencapsulation of a Bifidobacterium Strain with Starch as an Approach to Prolonging Viability during Storage. J. Appl. Microbiol..

[138] Morgan C.A., Herman N., White P.A., Vesey G. (2006). Preservation of Micro-Organisms by Drying: A Review. J. Microbiol. Methods.

[139] Jayaprakash P., Gaiani C., Edorh J.-M., Borges F., Beaupeux E., Maudhuit A., Desobry S. (2023). Comparison of Electrostatic Spray Drying, Spray Drying, and Freeze Drying for Lacticaseibacillus Rhamnosus GG Dehydration. Foods.

[140] Mattila-Sandholm T., Myllärinen P., Crittenden R., Mogensen G., Fondén R., Saarela M. (2002). Technological Challenges for Future Probiotic Foods. Int. Dairy J..

[141] Huang S., Vignolles M.L., Chen X.D., Le Loir Y., Jan G., Schuck P., Jeantet R. (2017). Spray Drying of Probiotics and Other Food-Grade Bacteria: A Review. Trends Food Sci. Technol..

[142] Huang S. Spray Drying of Probiotic Bacteria: From Molecular Mechanism to Pilot-Scale Productio. https://hal.science/tel-02791240/.

[143] Anal A.K., Singh H. (2007). Recent Advances in Microencapsulation of Probiotics for Industrial Applications and Targeted Delivery. Trends Food Sci. Technol..

[144] Wang X., Xie W., Zhang S., Shao Y., Cai J., Cai L., Wang X., Shan Z., Zhou H., Li J. (2022). Effect of Microencapsulation Techniques on the Stress Resistance and Biological Activity of Bovine Lactoferricin-Lactoferrampin-Encoding Lactobacillus Reuteri. Foods.

[145] Kiprono S., Wambani J., Langat V., Rono J., Yang G. (2024). Microencapsulation of Probiotics and Its Application as Co-Delivery Systems: Review of Literature. ES Food Agrofor..

[146] Kailasapathy K. (2002). Microencapsulation of Probiotic Bacteria: Technology and Potential Applications. Curr. Issues Intest. Microbiol..

[147] Rajam R., Subramanian P. (2022). Encapsulation of Probiotics: Past, Present and Future. Beni Suef Univ. J. Basic. Appl. Sci..

[148] Barajas-Álvarez P., González-Ávila M., Espinosa-Andrews H. (2023). Recent Advances in Probiotic Encapsulation to Improve Viability under Storage and Gastrointestinal Conditions and Their Impact on Functional Food Formulation. Food Rev. Int..

[149] Arslan-Tontul S., Erbas M., Gorgulu A. (2019). The Use of Probiotic-Loaded Single- and Double-Layered Microcapsules in Cake Production. Probiotics Antimicrob. Proteins.

[150] Malmo C., La Storia A., Mauriello G. (2013). Microencapsulation of Lactobacillus Reuteri DSM 17938 Cells Coated in Alginate Beads with Chitosan by Spray Drying to Use as a Probiotic Cell in a Chocolate Soufflé. Food Bioprocess Technol..

[151] Gul O. (2017). Microencapsulation of Lactobacillus Casei Shirota by Spray Drying Using Different Combinations of Wall Materials and Application for Probiotic Dairy Dessert. J. Food Process Preserv..

[152] Rutz J.K., Borges C.D., Zambiazi R.C., da Rosa C.G., da Silva M.M. (2016). Elaboration of Microparticles of Carotenoids from Natural and Synthetic Sources for Applications in Food. Food Chem..

[153] Papillo V.A., Locatelli M., Travaglia F., Bordiga M., Garino C., Arlorio M., Coïsson J.D. (2018). Spray-Dried Polyphenolic Extract from Italian Black Rice (Oryza Sativa L., Var. Artemide) as New Ingredient for Bakery Products. Food Chem..

[154] Adinepour F., Pouramin S., Rashidinejad A., Jafari S.M. (2022). Fortification/Enrichment of Milk and Dairy Products by Encapsulated Bioactive Ingredients. Food Res. Int..

[155] Rashidinejad A., Bahrami A., Rehman A., Rezaei A., Babazadeh A., Singh H., Jafari S.M. (2022). Co-Encapsulation of Probiotics with Prebiotics and Their Application in Functional/Synbiotic Dairy Products. Crit. Rev. Food Sci. Nutr..

[156] Amadoro C., Rossi F., Pallotta M.L., Gasperi M., Colavita G. (2018). Traditional Dairy Products Can Supply Beneficial Microorganisms Able to Survive in the Gastrointestinal Tract. LWT.

[157] Maciel G.M., Chaves K.S., Grosso C.R.F., Gigante M.L. (2014). Microencapsulation of Lactobacillus Acidophilus La-5 by Spray-Drying Using Sweet Whey and Skim Milk as Encapsulating Materials. J. Dairy Sci..

[158] Leylak C., Özdemir K.S., Gurakan G.C., Ogel Z.B. (2021). Optimisation of Spray Drying Parameters for Lactobacillus Acidophilus Encapsulation in Whey and Gum Arabic: Its Application in Yoghurt. Int. Dairy J..

[159] Fazilah N.F., Hamidon N.H., Ariff A.B., Khayat M.E., Wasoh H., Halim M. (2019). Microencapsulation of Lactococcus Lactis Gh1 with Gum Arabic and Synsepalum Dulcificum via Spray Drying for Potential Inclusion in Functional Yogurt. Molecules.

[160] Picot A., Lacroix C. (2004). Encapsulation of Bifidobacteria in Whey Protein-Based Microcapsules and Survival in Simulated Gastrointestinal Conditions and in Yoghurt. Int. Dairy J..

[161] de Andrade D.P., Bastos S.C., Ramos C.L., Simões L.A., de Andrade Teixeira Fernandes N., Botrel D.A., Magnani M., Schwan R.F., Dias D.R. (2023). Microencapsulation of Presumptive Probiotic Bacteria Lactiplantibacillus Plantarum CCMA 0359: Technology and Potential Application in Cream Cheese. Int. Dairy J..

[162] Borrás-Enríquez A.J., Delgado-Portales R.E., de-La Cruz-Martínez A., Delgado-Portales R.E., González-Chávez M.M., Abud-Archila M., Moscosa-Santillán M. (2018). Microbiological-Physicochemical Assessment and Gastrointestinal Simulation of Functional (Probiotic and Symbiotic) Gouda-Type Cheeses during Ripening. Rev. Mex. Ing. Quim..

[163] Sharifi S., Rezazad-Bari M., Alizadeh M., Almasi H., Amiri S. (2021). Use of Whey Protein Isolate and Gum Arabic for the Co-Encapsulation of Probiotic Lactobacillus Plantarum and Phytosterols by Complex Coacervation: Enhanced Viability of Probiotic in Iranian White Cheese. Food Hydrocoll..

[164] Radulović Z., Miočinović J., Mirković N., Mirković M., Paunović D., Ivanović M., Seratlić S. (2017). Survival of Spray-Dried and Free-Cells of Potential Probiotic Lactobacillus Plantarum 564 in Soft Goat Cheese. Anim. Sci. J..

[165] Favaro-Trindade C., Okuro P.K., Eustáquio De Matos Junior F., Sílvia Favaro-Trindade C. (2013). Technological Challenges for Spray Chilling Encapsulation of Functional Food Ingredients. Food Technol. Biotechnol..

[166] Consoli L., Grimaldi R., Sartori T., Menegalli F.C., Hubinger M.D. (2016). Gallic Acid Microparticles Produced by Spray Chilling Technique: Production and Characterization. LWT.

[167] Figueiredo J.d.A., Silva C.R.d.P., Souza Oliveira M.F., Norcino L.B., Campelo P.H., Botrel D.A., Borges S.V. (2022). Microencapsulation by Spray Chilling in the Food Industry: Opportunities, Challenges, and Innovations. Trends Food Sci. Technol..

[168] Oxley J.D. (2012). Spray Cooling and Spray Chilling for Food Ingredient and Nutraceutical Encapsulation. Encapsulation Technologies and Delivery Systems for Food Ingredients and Nutraceuticals.

[169] Chalella Mazzocato M., Thomazini M., Favaro-Trindade C.S. (2019). Improving Stability of Vitamin B12 (Cyanocobalamin) Using Microencapsulation by Spray Chilling Technique. Food Res. Int..

[170] Arslan-Tontul S., Erbas M. (2017). Single and Double Layered Microencapsulation of Probiotics by Spray Drying and Spray Chilling. LWT-Food Sci. Technol..

[171] Pedroso D.d.L., Thomazini M., Heinemann R.J.B., Favaro-Trindade C.S. (2012). Protection of Bifidobacterium Lactis and Lactobacillus Acidophilus by Microencapsulation Using Spray-Chilling. Int. Dairy J..

[172] Rodrigues F.J., Cedran M.F., Bicas J.L., Sato H.H. (2020). Encapsulated Probiotic Cells: Relevant Techniques, Natural Sources as Encapsulating Materials and Food Applications—A Narrative Review. Food Res. Int..

[173] Bertoni S., Albertini B., Dolci L.S., Passerini N. (2018). Spray Congealed Lipid Microparticles for the Local Delivery of β-Galactosidase to the Small Intestine. Eur. J. Pharm. Biopharm..

[174] Gouin S. (2004). Microencapsulation: Industrial Appraisal of Existing Technologies and Trends. Trends Food Sci. Technol..

[175] Bampi G.B., Backes G.T., Cansian R.L., de Matos F.E., Ansolin I.M.A., Poleto B.C., Corezzolla L.R., Favaro-Trindade C.S. (2016). Spray Chilling Microencapsulation of Lactobacillus Acidophilus and Bifidobacterium Animalis Subsp. Lactis and Its Use in the Preparation of Savory Probiotic Cereal Bars. Food Bioprocess Technol..

[176] Silva M.P., Tulini F.L., Matos-Jr F.E., Oliveira M.G., Thomazini M., Fávaro-Trindade C.S. (2018). Application of Spray Chilling and Electrostatic Interaction to Produce Lipid Microparticles Loaded with Probiotics as an Alternative to Improve Resistance under Stress Conditions. Food Hydrocoll..

[177] Silva R., Pimentel T.C., Eustáquio de Matos Junior F., Esmerino E.A., Freitas M.Q., Fávaro-Trindade C.S., Silva M.C., Cruz A.G. (2022). Microencapsulation with Spray-Chilling as an Innovative Strategy for Probiotic Low Sodium Requeijão Cremoso Processed Cheese Processing. Food Biosci..

[178] Abu Elella M.H., Al Khatib A.O., Al-Obaidi H. (2024). Spray-Dried Nanolipid Powders for Pulmonary Drug Delivery: A Comprehensive Mini Review. Pharmaceutics.

[179] Favaro-Trindade C.S., de Matos Junior F.E., Okuro P.K., Dias-Ferreira J., Cano A., Severino P., Zielińska A., Souto E.B. (2021). Encapsulation of Active Pharmaceutical Ingredients in Lipid Micro/Nanoparticles for Oral Administration by Spray-Cooling. Pharmaceutics.

[180] Jacobsen C., García-Moreno P.J., Mendes A.C., Mateiu R.V., Chronakis I.S. (2018). Use of Electrohydrodynamic Processing for Encapsulation of Sensitive Bioactive Compounds and Applications in Food. Annu. Rev. Food Sci. Technol..

[181] Anu Bhushani J., Anandharamakrishnan C. (2014). Electrospinning and Electrospraying Techniques: Potential Food Based Applications. Trends Food Sci. Technol..

[182] Rostamabadi H., Assadpour E., Tabarestani H.S., Falsafi S.R., Jafari S.M. (2020). Electrospinning Approach for Nanoencapsulation of Bioactive Compounds; Recent Advances and Innovations. Trends Food Sci. Technol..

[183] Charles A.P.R., Jin T.Z., Mu R., Wu Y. (2021). Electrohydrodynamic Processing of Natural Polymers for Active Food Packaging: A Comprehensive Review. Compr. Rev. Food Sci. Food Saf..

[184] Roy S., Kumar R., Acooli A., Roy S., Chatterjee A., Chattaraj S., Nayak J., Jeon B.-H., Basu A., Banerjee S. (2024). Transforming Nanomaterial Synthesis through Advanced Microfluidic Approaches: A Review on Accessing Unrestricted Possibilities. J. Compos. Sci..

[185] Niamah A.K., Gddoa Al-Sahlany S.T., Ibrahim S.A., Verma D.K., Thakur M., Singh S., Patel A.R., Aguilar C.N., Utama G.L. (2021). Electro-Hydrodynamic Processing for Encapsulation of Probiotics: A Review on Recent Trends, Technological Development, Challenges and Future Prospect. Food Biosci..

[186] Martín M.J., Lara-Villoslada F., Ruiz M.A., Morales M.E. (2015). Microencapsulation of Bacteria: A Review of Different Technologies and Their Impact on the Probiotic Effects. Innov. Food Sci. Emerg. Technol..

[187] Zare M., Dziemidowicz K., Williams G.R., Ramakrishna S. (2021). Encapsulation of Pharmaceutical and Nutraceutical Active Ingredients Using Electrospinning Processes. Nanomaterials.

[188] Mendes A.C., Chronakis I.S. (2021). Electrohydrodynamic Encapsulation of Probiotics: A Review. Food Hydrocoll..

[189] Moayyedi M., Eskandari M.H., Rad A.H.E., Ziaee E., Khodaparast M.H.H., Golmakani M.-T. (2018). Effect of Drying Methods (Electrospraying, Freeze Drying and Spray Drying) on Survival and Viability of Microencapsulated Lactobacillus Rhamnosus ATCC 7469. J. Funct. Foods.

[190] Gomez-Mascaraque L.G., Morfin R.C., Pérez-Masiá R., Sanchez G., Lopez-Rubio A. (2016). Optimization of Electrospraying Conditions for the Microencapsulation of Probiotics and Evaluation of Their Resistance during Storage and In-Vitro Digestion. LWT-Food Sci. Technol..

[191] Škrlec K., Zupančič Š., Prpar Mihevc S., Kocbek P., Kristl J., Berlec A. (2019). Development of Electrospun Nanofibers That Enable High Loading and Long-Term Viability of Probiotics. Eur. J. Pharm. Biopharm..

[192] Feng K., Huang R., Wu R., Wei Y., Zong M., Linhardt R.J., Wu H. (2020). A Novel Route for Double-Layered Encapsulation of Probiotics with Improved Viability under Adverse Conditions. Food Chem..

[193] Zaeim D., Sarabi-Jamab M., Ghorani B., Kadkhodaee R. (2019). Double Layer Co-Encapsulation of Probiotics and Prebiotics by Electro-Hydrodynamic Atomization. LWT.

[194] López-Rubio A., Sanchez E., Wilkanowicz S., Sanz Y., Lagaron J.M. (2012). Electrospinning as a Useful Technique for the Encapsulation of Living Bifidobacteria in Food Hydrocolloids. Food Hydrocoll..

[195] Coghetto C.C., Brinques G.B., Siqueira N.M., Pletsch J., Soares R.M.D., Ayub M.A.Z. (2016). Electrospraying Microencapsulation of Lactobacillus Plantarum Enhances Cell Viability under Refrigeration Storage and Simulated Gastric and Intestinal Fluids. J. Funct. Foods.

[196] Moreno J.S., Dima P., Chronakis I.S., Mendes A.C. (2021). Electrosprayed Ethyl Cellulose Core-Shell Microcapsules for the Encapsulation of Probiotics. Pharmaceutics.

[197] Feng K., Huangfu L., Liu C., Bonfili L., Xiang Q., Wu H., Bai Y. (2023). Electrospinning and Electrospraying: Emerging Techniques for Probiotic Stabilization and Application. Polymers.

[198] Gómez-Mascaraque L.G., Ambrosio-Martín J., Perez-Masiá R., Lopez-Rubio A. (2017). Impact of Acetic Acid on the Survival of *L. Plantarum* upon Microencapsulation by Coaxial Electrospraying. J. Healthc. Eng..

[199] Yilmaz M.T., Taylan O., Karakas C.Y., Dertli E. (2020). An Alternative Way to Encapsulate Probiotics within Electrospun Alginate Nanofibers as Monitored under Simulated Gastrointestinal Conditions and in Kefir. Carbohydr. Polym..

[200] Coghetto C.C., Flores S.H., Brinques G.B., Záchia Ayub M.A. (2016). Viability and Alternative Uses of a Dried Powder, Microencapsulated Lactobacillus Plantarum without the Use of Cold Chain or Dairy Products. LWT-Food Sci. Technol..

[201] Nachal N., Moses J.A., Karthik P., Anandharamakrishnan C. (2019). Applications of 3D Printing in Food Processing. Food Eng. Rev..

[202] Yoha K.S., Anukiruthika T., Anila W., Moses J.A., Anandharamakrishnan C. (2021). 3D Printing of Encapsulated Probiotics: Effect of Different Post-Processing Methods on the Stability of Lactiplantibacillus Plantarum (NCIM 2083) under Static in Vitro Digestion Conditions and during Storage. LWT.

[203] Kuo C.-C., Clark S., Qin H., Shi X. (2022). Development of a Shelf-Stable, Gel-Based Delivery System for Probiotics by Encapsulation, 3D Printing, and Freeze-Drying. LWT.

[204] Zhang L., Lou Y., Schutyser M.A.I. (2018). 3D Printing of Cereal-Based Food Structures Containing Probiotics. Food Struct..

[205] Tomašević I., Putnik P., Valjak F., Pavlić B., Šojić B., Bebek Markovinović A., Bursać Kovačević D. (2021). 3D Printing as Novel Tool for Fruit-Based Functional Food Production. Curr. Opin. Food Sci..

[206] Racaniello G.F., Silvestri T., Pistone M., D’Amico V., Arduino I., Denora N., Lopedota A.A. (2024). Innovative Pharmaceutical Techniques for Paediatric Dosage Forms: A Systematic Review on 3D Printing, Prilling/Vibration and Microfluidic Platform. J. Pharm. Sci..

[207] Wang K., Huang K., Wang L., Lin X., Tan M., Su W. (2024). Microfluidic Strategies for Encapsulation, Protection, and Controlled Delivery of Probiotics. J. Agric. Food Chem..

[208] Qi P., Lv J., Yan X., Bai L., Zhang L. (2023). Microfluidics: Insights into Intestinal Microorganisms. Microorganisms.

[209] Liu H., Singh R.P., Zhang Z., Han X., Liu Y., Hu L. (2021). Microfluidic Assembly: An Innovative Tool for the Encapsulation, Protection, and Controlled Release of Nutraceuticals. J. Agric. Food Chem..

[210] Yang X., Nie W., Wang C., Fang Z., Shang L. (2024). Microfluidic-Based Multifunctional Microspheres for Enhanced Oral Co-Delivery of Probiotics and Postbiotics. Biomaterials.

[211] Logesh D., Vallikkadan M.S., Leena M.M., Moses J.A., Anandharamakrishnan C. (2021). Advances in Microfluidic Systems for the Delivery of Nutraceutical Ingredients. Trends Food Sci. Technol..

[212] Zhao C., Zhu Y., Kong B., Huang Y., Yan D., Tan H., Shang L. (2020). Dual-Core Prebiotic Microcapsule Encapsulating Probiotics for Metabolic Syndrome. ACS Appl. Mater. Interfaces.

[213] Luo Y., Ma Z., De Souza C., Wang S., Qiao F., Yi H., Gong P., Zhang Z., Liu T., Zhang L. (2024). Microfluidic Fabrication of Encapsulated Probiotic Microspheres Using Cysteine-Modified Chitosan with Dual Functions of Bacterial Adhesion and Intestinal Mucosal Adhesion. Food Hydrocoll..

[214] Quintana G., Gerbino E., Alves P., Simões P.N., Rúa M.L., Fuciños C., Gomez-Zavaglia A. (2021). Microencapsulation of Lactobacillus Plantarum in W/O Emulsions of Okara Oil and Block-Copolymers of Poly(Acrylic Acid) and Pluronic Using Microfluidic Devices. Food Res. Int..

[215] Singh P., Medronho B., Miguel M.G., Esquena J. (2018). On the Encapsulation and Viability of Probiotic Bacteria in Edible Carboxymethyl Cellulose-Gelatin Water-in-Water Emulsions. Food Hydrocoll..

[216] Tosi M.M., Ramos A.P., Esposto B.S., Jafari S.M. (2020). Dynamic Light Scattering (DLS) of Nanoencapsulated Food Ingredients. Characterization of Nanoencapsulated Food Ingredients.

[217] Sandoval-Castilla O., Lobato-Calleros C., García-Galindo H.S., Alvarez-Ramírez J., Vernon-Carter E.J. (2010). Textural Properties of Alginate–Pectin Beads and Survivability of Entrapped Lb. Casei in Simulated Gastrointestinal Conditions and in Yoghurt. Food Res. Int..

